# Role of Nuclear Non-Canonical Nucleic Acid Structures in Organismal Development and Adaptation to Stress Conditions

**DOI:** 10.3389/fgene.2022.823241

**Published:** 2022-02-23

**Authors:** Célia Alecki, Maria Vera

**Affiliations:** Department of Biochemistry, McGill University, Montreal, QC, Canada

**Keywords:** R-loop, DNA:RNA triplex, ncRNA, gene regulation, development, stress

## Abstract

Over the last decades, numerous examples have involved nuclear non-coding RNAs (ncRNAs) in the regulation of gene expression. ncRNAs can interact with the genome by forming non-canonical nucleic acid structures such as R-loops or DNA:RNA triplexes. They bind chromatin and DNA modifiers and transcription factors and favor or prevent their targeting to specific DNA sequences and regulate gene expression of diverse genes. We review the function of these non-canonical nucleic acid structures in regulating gene expression of multicellular organisms during development and in response to different stress conditions and DNA damage using examples described in several organisms, from plants to humans. We also overview recent techniques developed to study where R-loops or DNA:RNA triplexes are formed in the genome and their interaction with proteins.

## 1 Introduction

Non-canonical nucleic acid structures involving RNA can adopt different forms like R-loops and DNA:RNA triplexes and differ structurally from double-stranded DNA (dsDNA) by adopting a non-B-form (Wang et al., 1982; Radhakrishnan and Patel 1994). R-loops are composed of an RNA-DNA hybrid, a displaced single-stranded DNA (ssDNA), and a free RNA part that can be folded or not (Niehrs and Luke 2020). DNA:RNA triplexes are composed of dsDNA and an RNA strand wrapped around and contacting the DNA through Hoogsteen bonds (Li et al., 2016). Morgan and Wells first described non-canonical nucleic acid structures involving RNA in the 1960s (Morgan and Wells 1968). However, their *in vivo* identification and functions as regulatory elements have only been described during the last 2 decades (Ginno et al., 2012; Sentürk Cetin et al., 2019).

R-loops and DNA:RNA triplexes are dynamic and multifunctional structures. They regulate gene expression by inducing the activation or the repression of genes (Niehrs and Luke 2020; Li et al., 2016). The molecular mechanisms underlying their gene expression regulatory activities are diverse and involve different DNA sequences and protein partners, which are the focus of an extensive ongoing investigation. In this review, we discuss studies demonstrating that non-canonical nucleic acid structures act to provide sequence specificity to epigenetic and DNA modifications and transcription factors. They participate in the target of epigenetic modifiers, like the *Polycomb* complex PRC2, which lack sequence-specific binding activity, to specific regions of the genome and can also prevent the targeting of others (Alecki et al., 2020; Skourti-Stathaki et al., 2019; Arab et al., 2019; Grote et al., 2013; Schmitz et al., 2010; Postepska-Igielska et al., 2015; Cristini et al., 2018; Wang et al., 2018). They also favor or prevent the targeting of transcription factors (Sentürk Cetin et al., 2019; Arab et al., 2019; Cristini et al., 2018; Wang et al., 2018). Consequently, they can either repress or activate gene expression.

Non-canonical nucleic acid structures can act to orchestrate diverse gene expression regulatory mechanisms (Bacolla et al., 2015; Li et al., 2016; Crossley et al., 2019; Niehrs and Luke 2020). Therefore, they are considered important in complex biological processes like development and the response to stress conditions. Development requires a series of specific and synchronous events mediated by ubiquitously expressed transcription factors and epigenetic modifiers (Gilbert, 2000). The binding sites of transcription factors are present on the DNA at any time during development, and many of the epigenetic modifiers lack sequence-specific binding activity. Thus, additional cues must indicate which genes have to be expressed at specific developmental stages or cell types and which genes must be repressed. Non-canonical nucleic acid structures like R-loops and DNA:RNA triplexes can represent one of the missing cues to understand how such a precise regulation of gene expression occurs during development. Likewise, stress conditions impose fast changes in the transcriptional preferences of the cells. While most genes are repressed, specific non-coding and coding RNAs are upregulated to favor cell survival (Vihervaara et al., 2018). Our increased knowledge of the sequence, structure, dynamics, and function of non-canonical nucleic acid structures relies on developing tools that allow their identification *in vivo*. Hence, we review the biochemical and microscopy approaches utilized to study the non-canonical nucleic acid structure as well as the latest advances made to define their function and mechanisms of action in different organisms during development and stress conditions.

## 2 Techniques to Study the Expression of ncRNAs and Non-Canonical Nucleic Acid Structures

**FIGURE 1:**
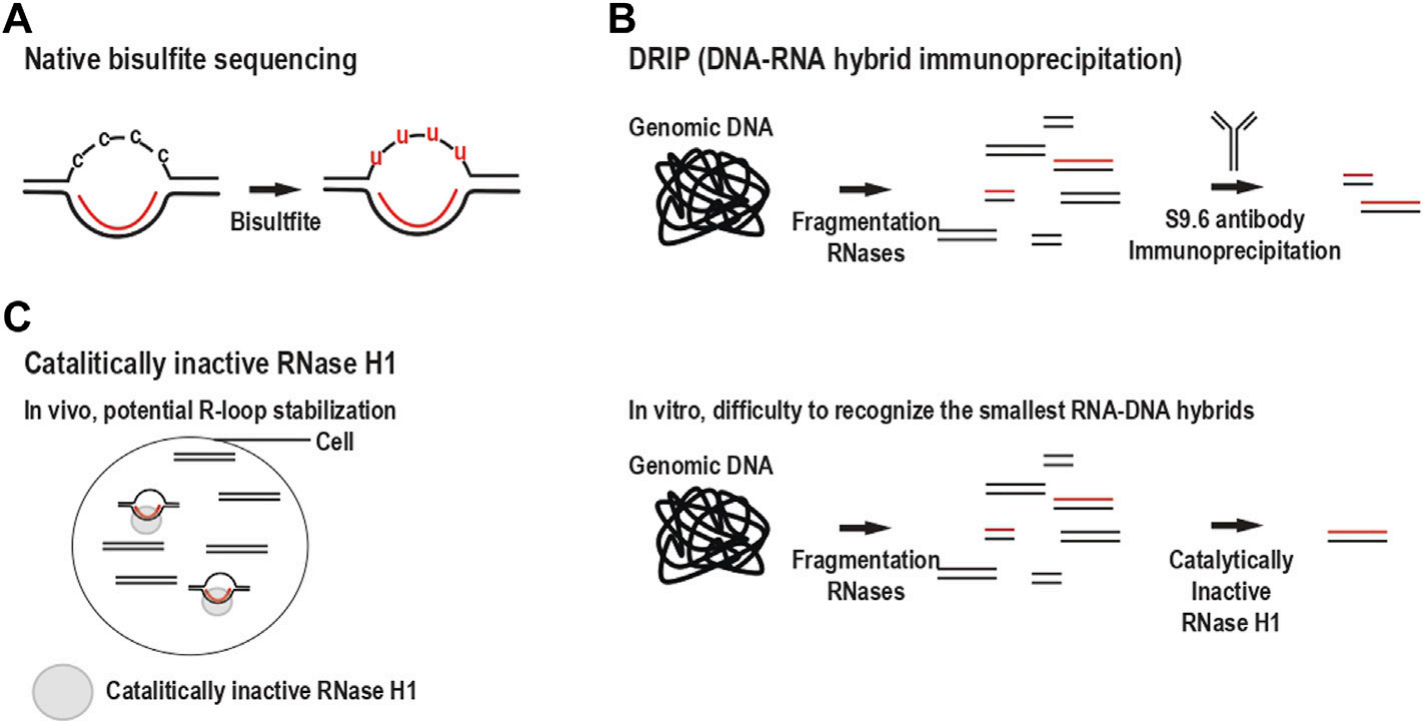
Schematic of the different techniques to identify sequences forming R-loops. **(A)** Bisulfite sequencing under native conditions. Bisulfite deaminates cytosines to uracils when present in the single strand of displaced DNA. After sequencing, the converted cytosines are used to identify the ssDNA part of an R-loop. **(B)** The immunoprecipitation of the RNA-DNA hybrids (DRIP) with the S9.6 antibody allows the detection of the RNA-DNA hybrids after extraction of the genomic DNA. Total nucleic acids are extracted from cells; these nucleic acids are fragmented and, ds and ssRNA are removed using RNases. RNA-DNA hybrid containing fragments are pulled down using S9.6 antibody and identified by sequencing. **(C)** The catalytically inactive RNase H1 enables the detection of the hybrid part of an R-loop *in vivo* (MapR) (left) or *in vitro* after extraction of the genomic DNA (DRIVE) (right). **[(C), Left]** The catalytically inactive RNase H1 is expressed in cells. It binds to accessible R-loops, cells are fixed, and inactive RNase H1 bound to RNA-DNA hybrid is immunoprecipitated, and RNA-DNA hybrids are identified by sequencing. **[(C), Right]** Similar to DRIP, nucleic acids are extracted from cells; they are fragmented and, ds and ssRNA are removed using nucleases. Because of its lower affinity than the S9.6 antibody, only the longer RNA-DNA hybrids are pulled down using a catalytically inactive RNase H1 and are identified by sequencing.

Over the last 2 decades, several sequencing techniques have been developed to annotate the ncRNA transcriptome, characterize their biogenesis, define their tissue and organism specificity, and elucidate the many fundamental functions they have to regulate gene expression (Sun and Chen 2020). The knowledge acquired by virtue of sequencing techniques is now being complemented with high-resolution microscopy to study the spatiotemporal regulation of ncRNA at the single-cell level (Figure 1) and biochemical approaches to characterize the formation and function of ncRNA engaged in non-canonical structures (Table 1). In this section, we review the principles and advantages that these techniques offer to non-canonical nucleic acid structures.

**TABLE 1:** Techniques allowing the identification of sequences forming R-loops.

Techniques		R-loop region targeted	+	−
Non-denaturing bisulfite-sequencing Yu et al. (2003)	Bisulfite	Displaced ssDNA	High resolution	Sensitive to DNA methylation Other non-canonical DNA structures exposed a ssDNA
DRIP Alecki et al. (2020); Hartono, (2018); Sanz and Chédin. (2019); Sanz et al. (2016); Wahba et al. (2016); Xu et al. (2020)	S9.6 antibody	RNA-DNA hybrid	Most widely used Optimized with multiple organisms Single-stranded version to determine the orientation of the R-loop A near base-pair resolution assay has been developed	Antibody bias against GC-rich abd longer sequences Nucleic acid extraction and harsh RNase treatment can affect the detection of the most unstable R-loops
DRIVE Ginno et al. (2012)	Catalytically inactive RNase H1 after nucleic acid extraction	RNA-DNA hybrid		RNase H1 ha an affinity for RNA-DNA hybrids 5 times lower compared to S9.6 antibody
R-ChIP (Chen et al. (2017), Chen et al. (2019), Skourti-Stathaki, Proudfoot et al. (2011)	Expression of catalytically inactive RNase H1 in cells	RNA-DNA hybrid	Can detect R-loop unstable R loops which are degraded during nucleic acid extraction	Stabilization of R-loop degraded by RNase H1 RNA-DNA hybrid has to be accessible

### 2.1 Biochemistry

Several techniques to detect non-canonical nucleic acid structures involving coding and non-coding RNA have developed over the past decades. In this section, we focus on R-loops (Table 1) and DNA:RNA triplexes and the main techniques utilized to identify RNA-RNA and RNA-protein interactions.

#### 2.1.1 R-Loops Detection

**D**NA-**R**NA hybrid **i**mmuno**p**recipitation (DRIP) is the primary method used to identify genome-wide RNA-DNA hybrids, the main component of an R-loop (Ginno et al., 2012) (Table 1). DRIP was developed in 2012 and relies on the recognition of RNA-DNA hybrids by the S9.6 antibody. The S9.6 antibody was first thought to recognize RNA-DNA hybrids without sequence specificity. However, a more detailed analysis has shown that it exhibits a bias toward GC-rich sequences and longer RNA-DNA hybrids (Phillips et al., 2013; König et al., 2017). S9.6 antibody also recognizes dsRNA with an affinity five times lower than RNA-DNA hybrids, forcing the removal of single- and double-stranded RNAs (ssRNA and dsRNA) with RNase A and RNase III treatments before nucleic acid fragmentation and immunoprecipitation and precluding the use of the S9.6 antibody for microscopy approaches due to the detection of RNA artifacts (Hartono, 2018; Smolka et al., 2021). DRIP relies on cell lysis and protein removal followed by total nucleic acid extraction with phenol-chloroform and ethanol precipitation. As a quality control to call peaks and identify areas in the genome rich in R-loops by DNA sequencing, a negative control sample is treated with RNase H (RNase H1 and H2) to remove RNAs from RNA-DNA hybrids. This basic DRIP protocol was first developed in Ntera2 mammalian cell lines and optimized to identify R-loop forming sequences in yeast (Wahba et al., 2016; Hartono, 2018), plant tissues (Xu et al., 2020), *Drosophila* embryos, and cell lines (Alecki et al., 2020)*.* Additionally, DRIP has been improved to determine the DNA strand specificity, using either the DNA or RNA moiety of the sample, and to increase the nucleotide resolution in sequencing experiments (Sanz et al., 2016; Sanz and Chédin 2019).

In 2020, a single-molecule assay was developed to obtain a near single-nucleotide resolution of the R-loop forming sequence (Malig and Chedin 2020). Unlike the previously mentioned protocol, this new method relies on non-denaturing bisulfite conversion of cytidine (C) to uridine (U) in the displaced single-stranded DNA followed by sequencing long-read, which identifies the entire R-loop and two mutually exclusive regions forming an R-loop (Malig and Chedin 2020) (Table 1). However, bisulfite converts any C to U in a single-stranded DNA. Therefore, other non-canonical nucleic acid structures that expose an ssDNA, like G-quadruplex or I-motif, can be detected. Hence, the specificity of bisulfite sequencing to detect R-loops should be validated by DRIP-sequencing. Although C methylation make ssDNA insensitive to bisulfite (Moye et al., 2015; Miglietta et al., 2020),R-loop and DNA methylation are incompatible in mammals and plants (Ginno et al., 2012; Xu et al., 2017) and R-loops have been involved in DNA demethylation via their recognition by the protein GADD45A, which recruit the demethylase TET1 (Arab et al., 2019).

Recently, R-loops were detected *in vivo* by expressing a catalytically inactive RNase H1 fused to the Micrococcal nuclease (MNase) (Yan and Sarma 2020). The expression of this chimeric protein leads to the cleavage of the DNA at the RNase H1 binding site. R-loops identified by this approach might be a minor fraction because RNase H1-MNase fusion protein would only bind R-loops that are protein-free and should outcompete the endogenous RNAse H1. Hence, this technique might be biased to identify stabilized R-loops because they are likely resolved by the endogenous RNase H1. Under physiological conditions, RNase H1 is present in the nucleus, but only a small fraction is bound to the DNA as RNase H1 is essentially targeted to chromatin under stress conditions (Lockhart et al., 2019). RNase H1 has two distinct RNA-DNA hybrid binding sites separated by multiple amino acids that have to bind, meaning that this approach will not be able to detect the smallest R-loops (Nowotny et al., 2008). Additionally, the catalytically inactive RNase H1 has a lower affinity for RNA-DNA hybrid than S9.6 antibody, making this technique less sensitive for detecting R-loops than DRIP (Ginno et al., 2012). In spite of these caveats, combining this technique with bisulfite conversion has made possible to provide strand specificity of the R-loops identified with MapR (Wulfridge and Sarma 2021).

#### 2.1.2 DNA:RNA Triplex Detection

DNA:RNA triplexes are composed of two DNA strands and one RNA in the major groove of the dsDNA formed by a Hoogsteen base pairing between the RNA and the purine-rich DNA strand (Li et al., 2016). The absence of antibodies or proteins that specifically recognize and bind these structures has made difficult their identification *in vivo*. The first protocol developed to discover DNA:RNA triplexes genome-wide (triplex capture assay) involves the incubation of biotinylated-RNA with isolated nuclei followed by streptavidin pull down to detect the DNA bound to the RNA (Postepska-Igielska et al., 2015). However, triplex capture assay creates artificial DNA:RNA triplexes, prevents the identification of endogenous triplexes, and does not distinguish DNA:RNA triplexes from hybrid G-quadruplexes or RNA-DNA hybrids. In 2019, Sentürk Cetin *et al.* developed other protocol to identify DNA:RNA triplexes *in vivo* in mammalian cells for the first time (Sentürk Cetin et al., 2019). Total nucleic acids are extracted using gentle lysis in the presence of proteinase K and RNA-DNA hybrids and ssRNA are removed by a short treatment with RNase H and RNase A, respectively. DNA- and RNA-associated DNA was immunoprecipitated with an antibody that recognizes DNA, then RNAs were eluted from the beads by digesting the DNA with DNase, and the RNA part of the triplexes were sequenced. In this protocol, samples are treated with a commercial RNase H for 30 min, which is insufficient to remove all RNA-DNA hybrids; thus, a fraction of the RNA-DNA hybrids, the main part of R-loops, might still be present and identified as DNA:RNA triplexes (Alecki and Francis 2021). As a result, they observed that 20% of the peaks of their putative DNA:RNA triplexes overlapped with previously published R-loop forming sequences, suggesting that these RNAs tend to form different non-canonical nucleic acid structures or that their protocol is not fully specific for the identification of DNA:RNA triplexes *in vivo*. Even so, this protocol allowed the identification of DNA:RNA triplexes *in vivo* for the first time, validating that the triplex capture assay using biotinylated RNA can be a tool to identify triplex-forming sequences.

#### 2.1.3 RNA-RNA Interactions

RNA molecules interact with other RNA molecules directly or through proteins. RNA-RNA interactions mediated by proteins are detected using cross-linking reagents, such as UV or formaldehyde. The RNAs are fragmented, and their extremities in proximity are ligated, and the RNA hybrids or chimeras are sequenced to identify RNA-RNA interactions (Gong et al., 2018; Lin and Miles 2019). The direct interaction between two RNA molecules is detected using the compound psoralen. Psoralens were used initially to identify RNA structure because they intercalate into dsRNAs. The subsequent cross-linking of dsRNAs with UV, removal of ssRNA by RNases, and ligation of dsRNA ends afford to sequence the chimeric RNAs (Lu and Chang 2018).

#### 2.1.4 RNA-Protein Interactions

RNA-protein interactions are commonly studied by RNA immunoprecipitation (RIP) and cross-linking immunoprecipitation (CLIP). CLIP relies on UV cross-linking before pulling down the protein of interest, followed by RNA sequencing in order to identify bound RNAs (Ramanathan et al., 2019). CLIP is also used to identify the specific nucleotide sequence bound by a protein. For this purpose, the unprotected RNA is digested with RNases, and protein-protected RNA analyze by sequencing (Hafner et al., 2021). However, the presence of post-translational modification can decrease the efficiency of UV cross-linking (Vieira-Vieira et al., 2021). Complementary techniques to CLIP have been developed to pull down the RNA and identify its interacting proteome by mass spectrometry. Most of these methods require the insertion of exogenous sequences, like the MS2 stem-loops, into the RNA sequence to facilitate its pull down with the cognate binding protein (Slobodin and Gerst 2010; Hafner et al., 2021). Alternatively, the exogenous expression of a modified CRISPR-Cas9 (clustered regularly interspaced short palindromic repeats-Cas) system where the inactive Cas9 fused to a BirA protein is directed to a specific mRNA and interacting proteins get labeled and recognized by BioID (Yi et al., 2020). A recently developed method identifies proteins at the proximity of a particular endogenous RNA without exogenous expression of factors or modification of RNAs (Yap et al., 2021). The RNA of interest is hybridized with digoxigenin-labeled antisense probes, and the digoxigenin is then bound by the so-called hybridization proximity (HyPro) enzyme. The RNAs and proteins in the proximity of the target mRNA are biotinylated by HyPro enzymes, pulled-down using streptavidin beads, and identified by RNA-seq and mass spectrometry, respectively. Even if this technique is limited to the RNA and protein interactome of a specific RNA, it may provide information on low abundant RNAs because it doesn’t require the immunoprecipitation step. A limitation of this method is the difficulty to discern between proximal or interacting proteins and RNAs. All techniques aiming to identify RNA-protein interactions lead to the detection of direct and indirect associations. In addition, these techniques are more qualitative than quantitative, and quantification only refers to a relative comparison between experimental conditions (Vieira-Vieira et al., 2021). Their main caveat is the limitation to a single RNA or protein and miss the complete picture of all cellular RNA-protein interactions.

### 2.2 Microscopy

Visualizing and analyzing the spatial distribution and dynamics of expression of ncRNA inside cells provide the means to relate their nuclear localization and expression regulation to their function. The spatiotemporal resolution obtained with microscopy approaches helps to study ncRNA expression and function in response to internal cues, like the cell-cycle regulation and cell-differentiation, and changes in their environment.

#### 2.2.1 Localization and Quantification of ncRNAs

Single-molecule fluorescence *in situ* hybridization (smFISH) is a robust technique widely used to quantify and localize single RNA molecules inside cells. smFISH was designed initially to detect mRNAs through the multimerization of specific antisense DNA oligonucleotides that are fluorescently labeled (Femino 1998). The localization of several fluorophores on an RNA molecule provides the sufficient signal-to-noise ratio to specifically detect the fluorescence signal from an mRNA, whose localization is later analyzed by specific computational frameworks, like Fishquant (Mueller et al., 2013). Given that an RNA molecule must accommodate several probes to be detected, smFISH has worked to detect, quantify the abundance, and analyze the distribution of long ncRNAs (Cabili et al., 2015). The distribution of long ncRNAs in the cell is diverse. Still, it shows a predominantly nuclear localization that appears as discrete nuclear aggregates in 20% of the cases, supporting their role in gene expression and chromatin structure. A simultaneous two-color smFISH has distinguished the long ncRNA accumulation at different nuclear locations and transcription sites. In this protocol, the second set of probes, labeled with a different spectral fluorophore, detect the transcription of a proximal divergent gene to the ncRNA or a gene known to be regulated by the ncRNA (Cabili et al., 2015).

Given the multimerization nature of smFISH, the original protocol had to be adapted for the specific detection of small ncRNAs, whose sequence is not long enough to accommodate several antisense probes. The single-molecule resolution for small ncRNAs has been accomplished by increasing the signal provided by fewer antisense oligonucleotides and branching DNA probes. Amplification of the signal provided by a single antisense oligonucleotide with a hapten by tyramide signal amplification (TSA), TSA-FISH. TSA signal amplification utilizes the enzymatic reaction of the horseradish peroxidase and fluorescently labels tyramide substrates that are deposited at the reaction site (Kwon 2013). In branched FISH (bDNA FISH), two antisense oligonucleotides hybridized the RNA and provided a linker sequence. The linker sequence is hybridized by an amplifier that provides a sequence to multimerized oligonucleotides that are recognized by fluorescently labeled probes (Battich et al., 2013; Sinnamon and Kevin 2014).

#### 2.2.2 Dynamics of ncRNA Expression

Visualizing RNAs in live cells to investigate their expression dynamics relies upon the same fluorescent multimerization principle that smFISH. The MS2 and PP7 are the two most successfully used systems for ncRNAs, like telomeric RNA TERRA (Cusanelli et al., 2013), the X-inactive specific transcript Xist , and the antisense *GAL10* ncRNA (Lenstra et al., 2015). The MS2 and PP7 systems are RNA aptamers derived from bacteriophages that are inserted as an array of 12 to 24 stem-loops in the gene encoding for the ncRNA. Each of the stem-loops is tightly bound by the cognate protein, MCP and PCP, respectively, fused to a fluorescent protein, like the green fluorescent protein (GFP) (Vera et al., 2016). The MS2 and PP7 have been combined to tag in the same cell an ncRNA and its regulated mRNAs to link ncRNA expression dynamics to their regulatory function on mRNA transcription (Lenstra et al., 2015). The caveats of the MS2 and PP7 systems to follow the expression of small ncRNAs is that they preclude their function by adding a long RNA sequence and a bulky protein load.

## 3 Non-Coding RNAs in Gene Expression Regulation

The expression of ncRNAs is more tissue-specific than the mRNAs; while 50% of ncRNAs are expressed in a maximum of two different tissues in humans, 65% of mRNAs are detected in the tested sixteen tissues (Derrien et al., 2012). Another characteristic of ncRNAs is their low abundance, as they are expressed at much lower levels than mRNAs. ncRNAs regulate gene expression by mainly four different mechanisms, 1) sequestering microRNAs (miRNAs), 2) targeting proteins to the chromatin, 3) *cis*-transcriptional regulation of a coding gene, and 4) forming non-canonical nucleic acid structures, RNA-loops, and DNA:RNA triplexes, to promote or prevent targeting transcription factors or chromatin and DNA modifiers.

(i) miRNAs have been widely studied over the past 25 years. They are small RNAs of 21 to 25 nucleotides that regulate gene expression at different steps from transcription to translation. They bind mRNAs in their 5′ or 3′ UTR to prevent translation and can also trigger mRNA degradation by forming an RNA duplex and recruiting the RISC complex (for review O'Brien et al., 2018). miRNAs can also interact with DNA promoting a favorable chromatin context and gene upregulation. The role of ncRNA in the regulation of miRNA availability has been extensively reviewed. It relies on their capacity to bind several miRNAs like a sponge and regulate their activity by competing to bind their target mRNA (for review Fernandes et al., 2019). (ii) Long ncRNAs target chromatin modifiers to the promoter of specific genes, which affect their expression (for review Statello et al., 2021). (iii) There is a set of genic long ncRNAs known as promoted associated ncRNAs (pancRNAs) that regulate in *cis* the transcription of coding genes. pancRNAs are the product of transcription of bidirectional promoters enriched in CpG islands, which drive the expression of two divergent transcripts, an mRNA and a stable ncRNA, the pancRNA (Chellini et al., 2020; An et al., 2021). The expression of pancRNA correlates with the expression of their divergent protein-coding gene, and they have been proposed to activate their transcription by promoting local epigenetic changes (Uesaka et al., 2014; Hamazaki et al., 2015). Before being referred to as pancRNAs, promoter-derived ncRNAs were known as promoter upstream transcripts (PROMPTs), promoter- associated long RNAs (PALRs), promoter-associated small RNAs (PASRs), upstream antisense RNAs (UaRNAs), stable unannotated transcripts (SUTs), and cryptic unstable transcripts (CUTs). They are mostly associated with the activation of transcription of the divergent mRNA (for review An et al., 2021). However, ncRNA originated from the intergenic RNA polymerase I (Pol I) promoter (pRNAs) overlap with the promoter of ribosomal RNAs (rRNAs), forming a DNA:RNA triplex that mediates the silencing of ribosomal genes (Santoro et al., 2010; Wehner et al., 2014; Vydzhak et al., 2020).

In this review, we refer to genome wide studies that have identified the position of R-loops and DNA:RNA triplexes and how they evolved in response to developmental or stress cues. We also provide mechanistic examples that highlight the role of ncRNAs forming DNA:RNA triplex and R-loops in regulating gene expression by targeting proteins to the chromatin during development and stress conditions. Four out of the dozen of mechanistic examples we overview concern Polycomb group proteins targeting by R-loop during development, or triplexes during development or stress. PRC2 complex is an essential epigenetic regulator conserved from plants to mammals. It lacks sequence-specific DNA binding activity but its targeting to the genome is not random. PRC2 complex binds to thousand of coding and non-coding RNAs (Kaneko et al., 2013; Khalil et al., 2009; Zhao et al., 2010), some forming non-canonical nucleic acid structures (Mondal et al., 2015, 3; Grote and Herrmann 2013; Skourti-Stathaki et al., 2019)*,* and PRC2-target sites form R-loop in *Drosophila melanogaster* (Alecki et al., 2020).

## 4 Non-Canonical RNA Structures in Organism Development

### 4.1 R-Loops and Development

#### 4.1.1 Where They Are Formed and Their Implications for the Organism

Genome-wide studies have identified R-loops during the development of several organisms. R-loops have been observed in *Drosophila melanogaster* embryos at two different stages of development: at two to 6 h (h) that corresponds to the steps of cellularization and gastrulation, and at 10–14 h during the embryo segmentation (Alecki et al., 2020). The study was restricted to detect R-loops in DNA elements called *Polycomb* response elements (PREs). PREs can be transcribed into ncRNAs and form R-loops. A subset of R-loops formed in PREs in 2–6 h old embryos was absent in 10–14 h embryos. PREs covered by R-loops had a higher level of the repressive transcription mark H3K27me3 than PREs lacking R-loops, and PREs which have formed an R-loop only in 2–6 h old embryos are more likely to be repressed by *Polycomb* group proteins in 10–14 h old embryos. *Polycomb* group trimethylated the lysine 27 of histone 3 (H3K27me3) and bound to it to compact the chromatin and inhibit its remodeling. Hence, this result suggested that forming an R-loop at PRE sequences during an early developmental stage may lead to *Polycomb-*dependent gene repression at a later stage. Their data shows that only a small number of R-loops are exclusively formed in 2–6 h embryos, but a higher number of them formed in early embryos and showed a decrease in the R-loop level in 10–14 h embryos, suggesting that upon development and cell differentiation some cells form R-loop and some other don’t.

R-loops forming at PREs are not the only developmentally regulated R-loops in *Drosophila*. *Drosophila* embryos also form R-loops in retrotransposon sequences known as Long interspersed nuclear elements (LINE). R-loops are enriched in LINEs and retrotransposons in embryos but S2 cells (derived from 20 to 24 h old embryos) have them enriched at low complexity sequences and simple repeats (Zeng et al., 2021). The formation of R-loops over LINE sequences was previously reported in the human LINE-1 sequence, and it is related to the Aicardi-Goutières syndrome, an inherited encephalopathy that affects newborn infants. The Aicardi-Goutières syndrome is associated with mutations in the exonucleases TREX1 or SAMHD1, the RNA deaminase ADAR1R, and the RNase H2 (Benitez-Guijarro et al., 2018). All these mutations lead to an increase of R-loop levels and DNA hypomethylation, a decrease of LINE-1 retrotransposition, which result in an induction of the innate immune response and neurological dysfunction (Lim et al., 2015). RNase H2 activity is also required to degrade the RNA part of the RNA-DNA hybrid intermediate and complete the retrotransposition, suggesting that R-loop formation and resolution are two essential steps of transposon insertion (Benitez-Guijarro et al., 2018). R-loops misregulation has also been linked to myelodysplastic syndrome, a blood cell cancer in which blood cells don’t mature (Sperling et al., 2017). The myelodysplastic syndrome can be caused by a decrease of the DEAD-box protein DDX41, a putative RNA helicase that was shown to interact with RNA-DNA hybrids (Wang et al., 2018). The reduction of DDX41 leads to an increase of R-loops in hematopoietic stem and progenitor cells and increases inflammatory signaling, leading to a rise in the number of hematopoietic stem and progenitor cells (Weinreb et al., 2021).

These studies, together with the fact that R-loops are detected at repetitive Satellite sequences in mammalian cells (Zeng et al., 2021), suggest that R-loop dynamics during mammalian development might support gene expression changes during this process. Mouse embryonic stem cells (mESC) have been used to identify R-loop interactors using immunoprecipitation with the S9.6 antibody followed by mass spectrometry (Wu et al., 2021). RBP1, a subunit of RNA polymerase II, and DHX9, RNA helicase, were among the already known R-loop interactors and regulators identified in this study. Additionally, nucleolar proteins and proteins involved in rRNA transcription and processing were particularly enriched. This result was expected because the nucleolus is one of the hotspots for R-loop formation (Sollier et al., 2014). Some proteins of the DEAD-box family, which are known to be involved in R-loop regulation and bind RNAs were also enriched (Cargill et al., 2021). Interestingly, the knockdown of several members of the DEAD-box family leads to the overexpression of genes involved in cell fate commitment and neuronal differentiation (Wu et al., 2021), suggesting that the R-loops regulated by these proteins may be important for the pluripotency of mESC.

The idea that R-loop regulation is essential for the pluripotency of stem cells is further supported by experiments done to reprogram Mouse Embryonic Fibroblast (MEFs) into pluripotent stem cells (PSC) (Li et al., 2020). During the dedifferentiation of MEFs using the Yamanaka cocktail, R-loop forming sequence changes preceded gene expression changes (Li et al., 2020). Interestingly, the stabilization of R-loops by knocking down RNase H1 using shRNA or expressing a catalytically inactive RNase H1 decreased or prevented MEF reprogramming, respectively. Genes involved in somatic cell state had increased R-loop levels upon the expression of the catalytically inactive RNase H1 in MEFs, which required a higher amount of the Yamanaka factor Sox2 to restore pluripotency. Surprisingly, Sox2 did not reduce R-loops’ total level but induced R-loops formation at other genes, including pluripotency genes. Sox2 interacts with Ddx5, a member of the DEAD-box family, and Dhx9, an RNA helicase, and inhibits their R-loop resolution activity. Overall, (Yaoyi et al., 2020) have highlighted the relevance of the tight regulation of R-loops to maintain the cell somatic state or reprogram pluripotency.

R-loop formation also coordinates different aspects of plant development. One study in *Arabidopsis thaliana* and another in rice have looked at R-loop formation genome-wide in various tissues and under different growth conditions (Fang et al., 2019; Xu et al., 2020). Differences in R-loop formation were found between seeds and flowers in *Arabidopsis thaliana* and seed and leaf callus in rice, where 30% of the R-loop forming sequences were different. Although the expression of ncRNA was not investigated, differences in R-loop formation between seeds and developed plants suggested that R-loops are developmentally regulated and, therefore, differentially expressed ncRNAs are probably involved in the formation of these non-canonical nucleic acid structures. Hence, efforts done over the last decade have identified R-loop forming sequences genome-wide in multiple organisms.

#### 4.1.2 R-Loops and the Regulation of Gene Expression: Mechanistic Examples

**FIGURE 2:**
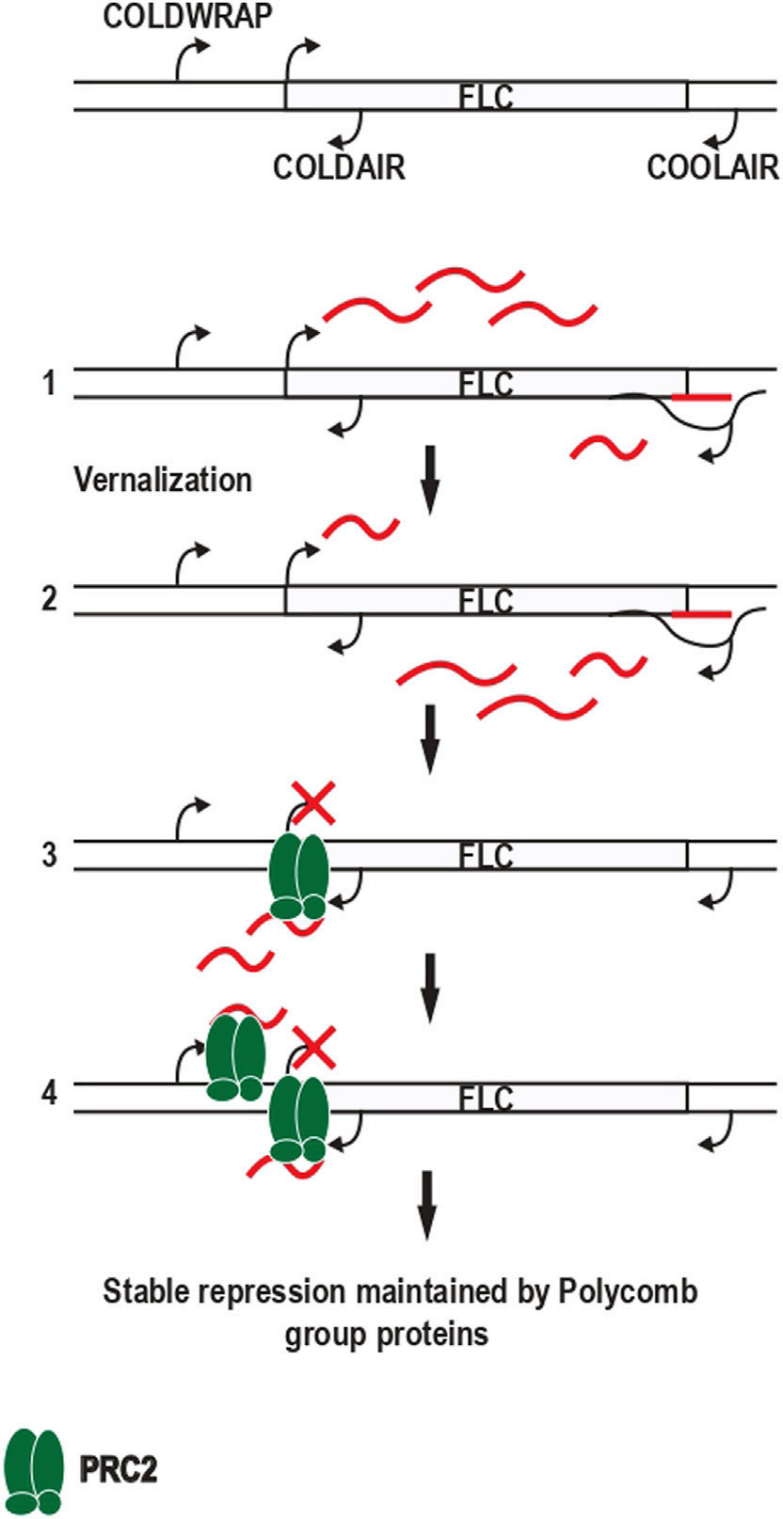
Repression of the FLC locus following vernalization involving ncRNAs and PRC2. Above is a schematic of the FLC locus, which is composed of the FLC gene and three ncRNA COOLAIR, COLDAIR, and COLWRAP. **(1)** Before vernalization FLC is expressed as well as COOLAIR in its short form forming an R loop. **(2)** Following vernalization, the expression of COOLAIR increases, and its long-form is produced, decreasing the expression of FLC. **(3)** The expression of COLDAIR allows the recruitment of PRC2, which trimethylates the lysine 27 of histone 3 and spreads along the chromatin, leading to the stable repression of FLC. **(4)** COLDWRAP expression enhances PRC2 recruitment and FLC repression.

Recent studies have started to highlight the role of R-loops in regulating the expression of ncRNAs and transcription factors with a role in development. There are two examples, one in plants and another in mammalian cell lines, that have been well documented. Among R-loops whose expression changed between the vegetative and reproductive state in plants, one of the best studied examples is in *Flowering locus C* (FLC) gene. In order to flower only after winter, plants like *Arabidopsis thaliana* need to repress the *FLC* gene (Costa and Dean 2019). Long cold exposure during vernalization leads to the progressive epigenetic silencing of the FLC gene. Three ncRNAs are transcribed from the FLC locus: COOLAIR, COLDAIR, and COLDWRAP. COOLAIR is a set of ncRNA antisense transcripts that originate close to the termination region of FLC. COOLAIR forms an extensive R-loop covering the FLC 3′ region (Sun et al., 2013), which in warm conditions triggers chromatin modifications that decrease transcriptional output from the whole locus. The R-loop is resolved by 3′ processing factors terminating COOLAIR transcription, in a process involving m^6^A methylation (Xu et al., 2021) (Figure 2). The R-loop is also important after 1–2 weeks of cold exposure where COOLAIR expression increases and clouds of nascent transcript form around the locus. This leads to a decrease of FLC expression because of their mutually exclusive production (Rosa et al., 2016). After 20 days of vernalization, COLDAIR, sense ncRNA transcribed from the first intron of FLC, is expressed. It is reported to interact with Polycomb complex PRC2 at the FLC locus (Kim et al., 2017). There is an initial nucleation site of PRC2 which tri-methylates the lysine 27 of histone 3, spread along the chromatin leading to stable repression of FLC (Yang et al., 2017). Finally, COLDWRAP, sense ncRNA transcribed in the promoter region of FLC, is expressed after COLDAIR and can serve as a backup mechanism in the absence of COLDAIR, allowing the Polycomb-dependent repression of FLC following transfer back to warm (Kim and Sung 2017). The promoter of FLC and its first intron form a gene loop identified by 3C depending on ncRNA COLDAIR and COLDWRAP (Kim and Sung 2017). The loop formation is thought to reinforce recruitment of PRC2 and its epigenetic mark H3K27me3. A second loop between the FLC promoter and termination site associates with FLC gene activity and this is lost as plants are transferred to cold (Crevillén et al., 2013).

**FIGURE 3:**
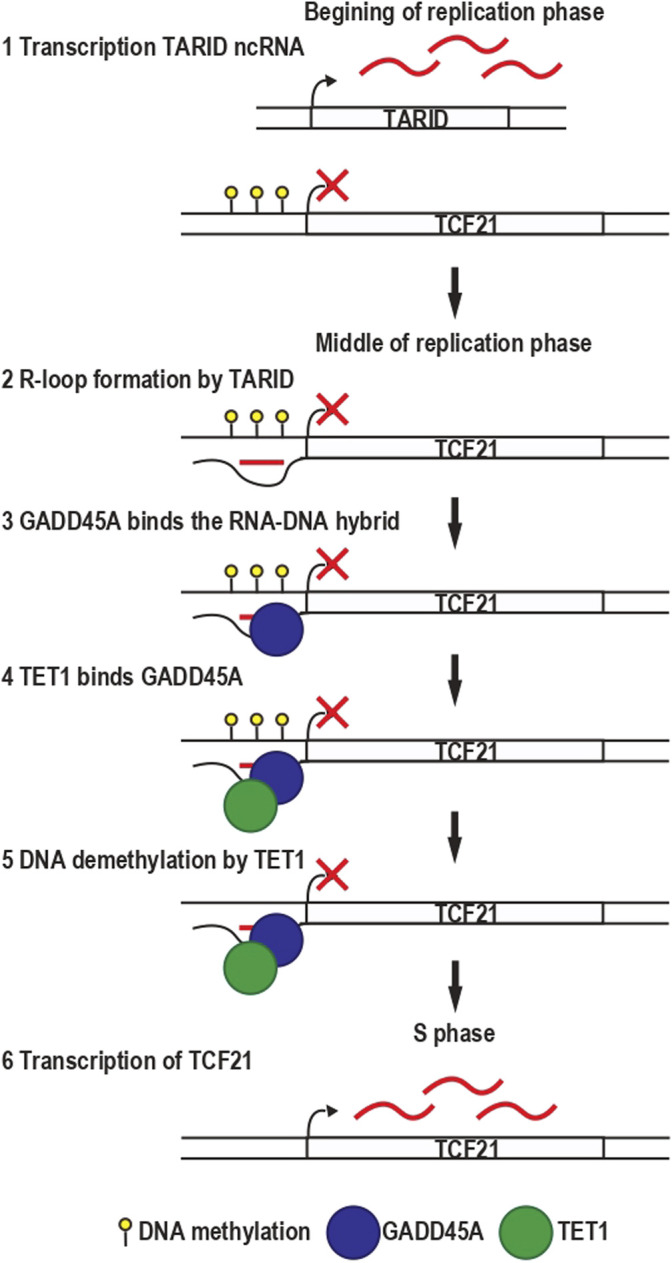
Transcription of the transcription factor *TCF21* gene is controlled by the R-loop formed by TARID ncRNA on its promoter. **(1)** TARID ncRNA is expressed at the beginning of the replication phase while TCF21 gene is repressed. **(2)** In the middle of the replication phase, TARID forms an R-loop in the promoter of *TCF21* gene. **(3)** This R-loop recruits GADD45A protein, **(4)** which in turn recruits TET1 to demethylate the DNA. **(4–6)** TCF21 promoter is demethylated, and the gene is transcribed in S phase.

The transcription factor TCF21 plays a critical role in developing several cell types during embryogenesis (Lotfi et al., 2021). The expression of the *TCF21* gene in human embryonic cells is controlled by an R-loop formed by the lncRNA TARID in the promoter of *TCF21* gene (Figure 3). The RNA-DNA hybrid part of this R-loop is recognized by the growth arrest and DNA-damage-inducible protein GADD45 alpha (GADD45A) protein, which interacts with the enzyme Ten-eleven translocation methylcytosine dioxygenase 1 (TET1). TET1 mediates the DNA demethylation of *TCF21* promoter, leading to its expression (Arab et al., 2019). Interestingly, all the previously mentioned events occur following DNA replication in a specific order during the cell cycle. DNA replication leads to the eviction of nucleosomes and their distribution between the two new DNA molecules. Likewise, the level of DNA methylation is divided by two and must be restored to maintain gene expression. Transcription of TARID starts at the beginning of the replication phase, and it is followed by R-loop formation, which recruits GADD45A and DNA demethylation to the *TCF21* promoter in the middle of the replication phase and leads to the transcription of *TCF21* during the S phase. This R-loop dependent targeting of TET1 is not restricted to the *TCF21* gene because the overexpression of RNase H1, enzyme degrading the RNA moiety of an RNA-DNA hybrid, leads to a decreased binding of GADD45A and TET1 at other binding sites controlling the expression of transcriptional coactivators and repressors, and chromatin binding proteins. On the contrary, knockdown of RNase H1 induces the stabilization of the R-loop and an increase in the general DNA methylation level (Arab et al., 2019).

### 4.2 DNA:RNA Triplexes and Development

#### 4.2.1 Where They Are Formed and Their Implications for the Organism

Both RNA and DNA involved in DNA:RNA triplexes have been identified in human cells independently (Sentürk Cetin et al., 2019). As expected, more than 50% of the RNAs involved in DNA:RNA triplex formation come from genes, and 8% of these structures are composed of ncRNAs. Likewise, 50% of the DNA sequences involved in DNA:RNA triplexes correspond to coding genes and 8% to ncRNAs. DNA and RNA involved in triplex are present in transcription start sites and enriched with active epigenetic marks H3K4 methyl. Surprisingly, the RNA part but not the DNA of DNA:RNA triplexes are enriched at super-enhancer, which are transcribed bidirectionally into ncRNAs (Xiao et al., 2021), suggesting that this RNA transcribed from enhancer can form triplexes in *trans*. Hence, it was proposed that these ncRNAs produced at enhancers might contact distal regions via the formation of DNA:RNA triplexes and induce transcription (Sentürk Cetin et al., 2019). However, the examples for which mechanistic details are available involved gene repression by DNA:RNA triplex.

#### 4.2.2 DNA:RNA Triplex and the Regulation of Gene Expression

**FIGURE 4:**
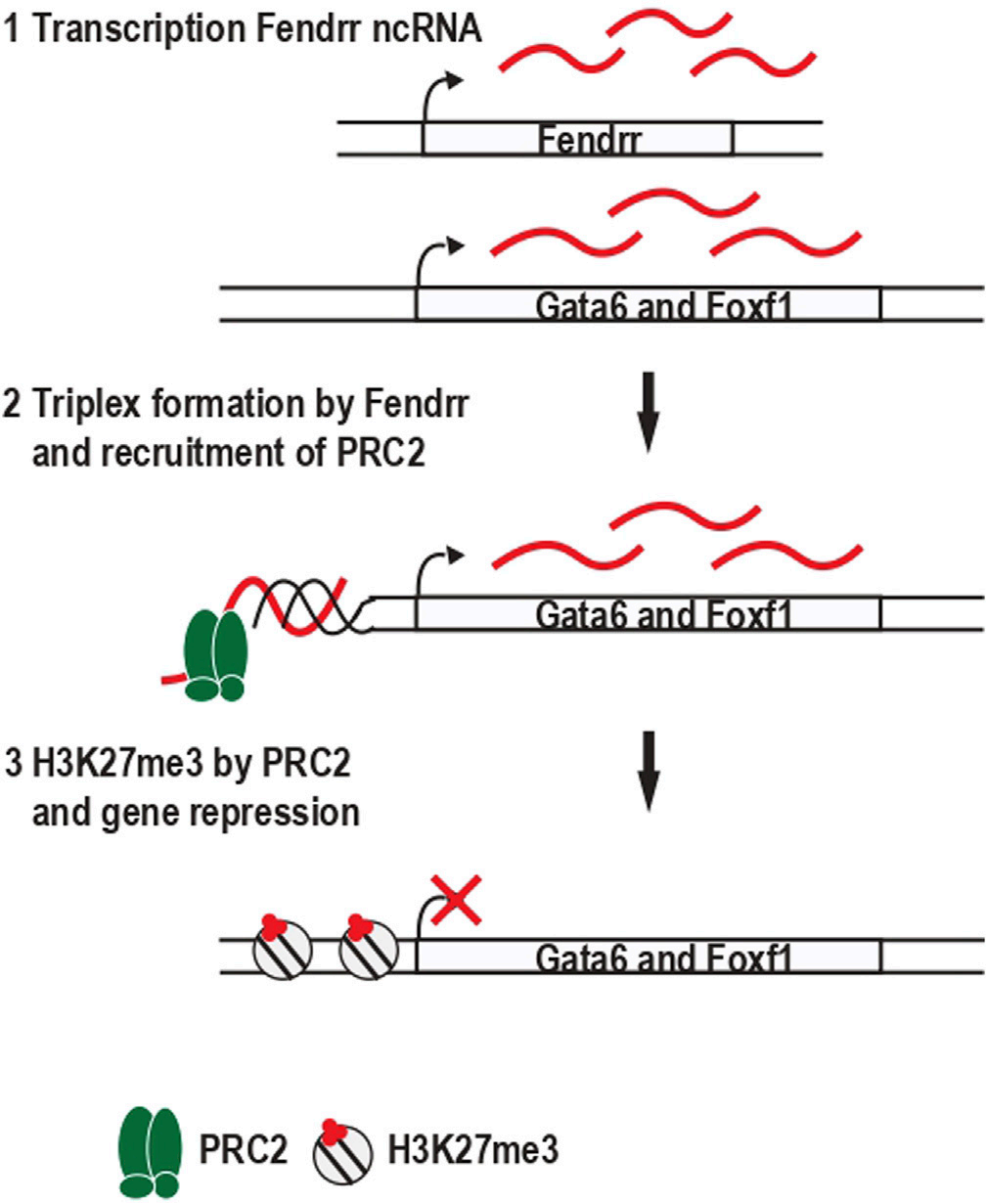
Repression of the transcription factor Gata6 and Foxf1 is mediated by a DNA:RNA triplex formed by the Fendrr ncRNA. **(1)** Fendrr is a ncRNA expressed in the mesoderm **(2)** Fendrr binds the *Polycomb* complex PRC2 and form an DNA:RNA triplex in the promoter of the genes Gata6 and Foxf1. **(3)** PRC2 trimethylates the lysine 27 of histone 3 and induces the stable repression of Gata6 and Foxf1 genes.

Two examples highlight the relevance of ncRNAs forming DNA:RNA triplexes during development. Fendrr is an essential ncRNA expressed in the mesoderm, whose depletion leads to embryonic death (Grote et al., 2013). The depletion of Fendrr leads to a decrease of the repressive mark H3K27me3 and an increase of the active mark H3K4me3 in the promoter region of transcription factor like Foxf1 (Grote et al., 2013). Fendrr interacts with the Polycomb complex PRC2, which trimethylates the lysine 27 of histone 3 promoting stable gene repression (Khalil et al., 2009). Using Chromatin Isolation by RNA purification (ChIRP)-seq analysis, it was shown the interaction of Fendrr with the promoter region of Foxf1 and Gata6, in a manner non-sensitive to RNase H, suggesting that Fendrr form an DNA:RNA triplex (Grote and Herrmann 2013) (Figure 4). The authors proposed a model in which Fendrr ncRNA bound by PRC2 includes an DNA:RNA triplex in the promoter region of its target genes, allowing the sequence-specific recruitment of PRC2, the deposition of H3K27me3, and the stable gene repression necessary for the proper embryonic development.

**FIGURE 5:**
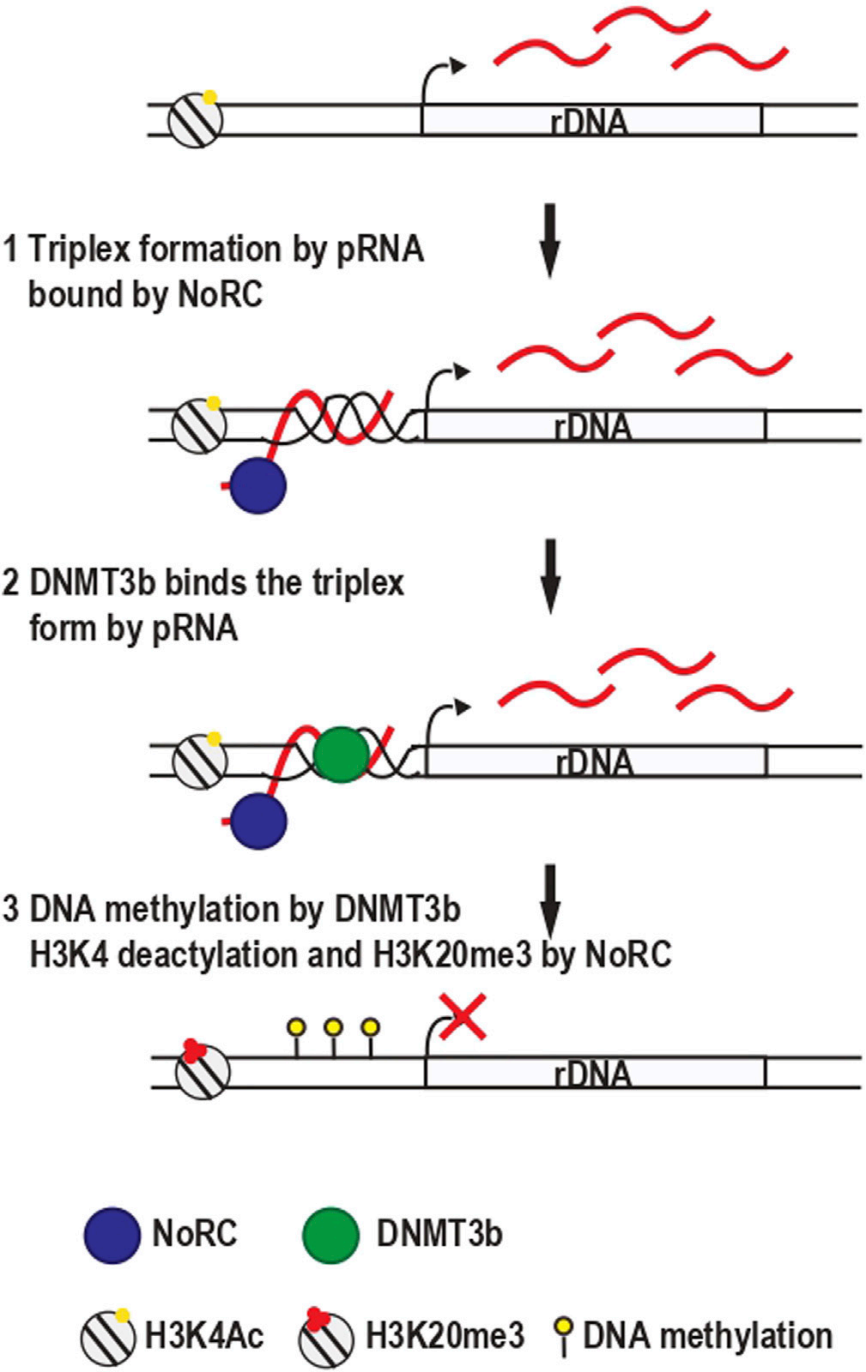
The promoter ncRNAs pRNA silence rDNA by forming a DNA:RNA triplex. rDNA genes are expressed and marked by the active epigenetic mark H3K4Ac. **(1)** pRNA transcribed from the PolI prmoter and degraded into ∼250 nts fragments and are complement to the rDNA promoter. pRNAs bound to NoRC complex form an DNA:RNA triplex in the promoter of actively transcribed rDNA genes. **(2)** The triplex induces the recruitment of the methyltransferase DNMT3b. **(3)** DNMT3b methylates the DNA, NoRC participates in the deacetylation of the lysine 4 and the trimethylation of the lysine 20 of the histone 3, inducing the stable repression of rDNA genes.

Promoter-associated RNAs, pRNAs, of ribosomal genes (pRNAs) are ncRNAs transcribed from the PolI promoter located in the intergenic region of ribosomal DNA (rDNA) (Vydzhak et al., 2020; Wehner et al., 2014; Santoro et al., 2010). These ncRNAs are degraded to ∼250 nucleotides long fragments that are bound by the chromatin remodeling complex NoRC (Vydzhak et al., 2020). pRNAs can bind the promoter sequence of rDNA by sequence complementation. The structure formed by pRNA and its target DNA is not sensitive to RNase H, nor RNase VI indicating that it is not composed of RNA-DNA hybrid nor dsRNA, respectively, and is not mediated by protein. Thus it is likely an DNA:RNA triplex as the EMSA (electrophoretic mobility shift assay) with dsDNA and pRNAs suggested (Schmitz et al., 2010). The formation of the DNA:RNA triplex between pRNAs and the promoter of rDNA leads to the targeting of the methyltransferase DNMT3b, which methylates the DNA (Schmitz et al., 2010) (Figure 5). NoRC binds pRNAs by recognizing a stem-loop structure, and this binding is essential for NoRC targeting to the nucleolus (Mayer et al., 2008). NoRC complex is then implicated in the deacetylation of histone H4 and the trimethylation of H3K20 an epigenetic repressive mark, allowing the stable repression of rDNA genes (Guetg et al., 2010).

More examples for DNA:RNA triplexes in development are expected, given recent findings in cell lines in culture. Taking advantage of the technique developed by Sentürk Cetin that identifies both the DNA and RNA part of DNA:RNA triplexes, it has been found that the ncRNA NEAT1 has two regions able to form DNA:RNA triplex *in vivo*. One of the triplex-forming regions was identified in HeLa S3 cells and the other in U2OS, suggesting that multiple domains from a single RNA can form triplex and their formation can be developmentally regulated or, at least, cell type-specific (Sentürk Cetin et al., 2019). ncRNA NEAT1 was shown to bind DNA and regulate gene expression by binding transcription factors and preventing them from being targeted to their binding sites. It can also bind and recruit PRC2 to repress specific genes. Finally, NEAT1 is also involved in RNA splicing and RNA stability (Wang et al., 2020, 1). The pull-down of NEAT1 RNA after incubation with protein-free DNA reveals that this ncRNA can bind multiple DNA sequences suggesting that NEAT1 can act *in trans* to target proteins by forming DNA:RNA triplexes (Sentürk Cetin et al., 2019). The research done so far to investigate the mechanism by which DNA:RNA triplexes operate during development shows that they mediate the modification of the DNA and chromatin that lead to the repression of protein-encoding and ribosomal genes.

### 4.3 ncRNA-mRNA Interaction

Interaction between one ncRNA and an mRNA has also been demonstrated to target proteins to specific loci. Hox genes are a family of conserved and essential transcription factors that are master regulators of the development of the anteroposterior axis (Hajirnis and Mishra 2021). Discovered in *Drosophila melanogaster*, they are organized in clusters, and their mutation can lead to homeotic transformation: the replacement of one segment by another. These genes have been duplicated in mammals and are present in four clusters on four different chromosomes. Their expression is controlled in a spatiotemporal manner and is essential for proper development (Mallo et al., 2010). In order to express Hox genes only in the appropriate compartments, these genes are repressed by epigenetic modifiers the *Polycomb* group proteins. The *Polycomb* complex PRC2 is a methyltransferase that trimethylates the lysine 27 of histone 3 (H3K27me3), and this epigenetic mark participates in the targeting of another *Polycomb* complex PRC1 which can compact the chromatin and stably repress gene expression (Steffen and Ringrose 2014). HOTAIR, ncRNA transcribed between the Hox genes HoxC11 and HoxC12 in mammals, is essential for the targeting of PRC2 *in trans* to the locus HoxD (Rinn et al., 2007; Gupta et al., 2010). In the absence of HOTAIR, PRC2 is not recruited to HoxD, leading to a derepression of this cluster and homeotic transformations (Gupta et al., 2010; Li et al., 2013; Tsai et al., 2010). Recently, Balas et al. showed that HOTAIR ncRNA hybridizes with Junctional Adhesion Molecule 2 (JAM2) mRNA. The formation of this dsRNA structure leads to a conformational change of HOTAIR RNA, decreasing the affinity of PRC2 for this RNA (Balas et al., 2020). They proposed that PRC2 binds HOTAIR and targets it to the HoxD cluster; the change of conformation upon hybridization with JAM2 leads to the release of PRC2 at its target site (Balas et al., 2020). Future investigations will determine whether JAM2, transcribed from a different chromosome than HoxD, forms a non-canonical nucleic acid structure at the locus HoxD, which will also explain the specific targeting of PRC2.

The results obtained over the past decade allow us to better-understanding how ncRNAs act during development to regulate gene expression directly or indirectly via the recruitment of proteins.

## 5 Non-Canonical Nucleic Acid Structures and Their Role in Stressed Cells

**FIGURE 6:**
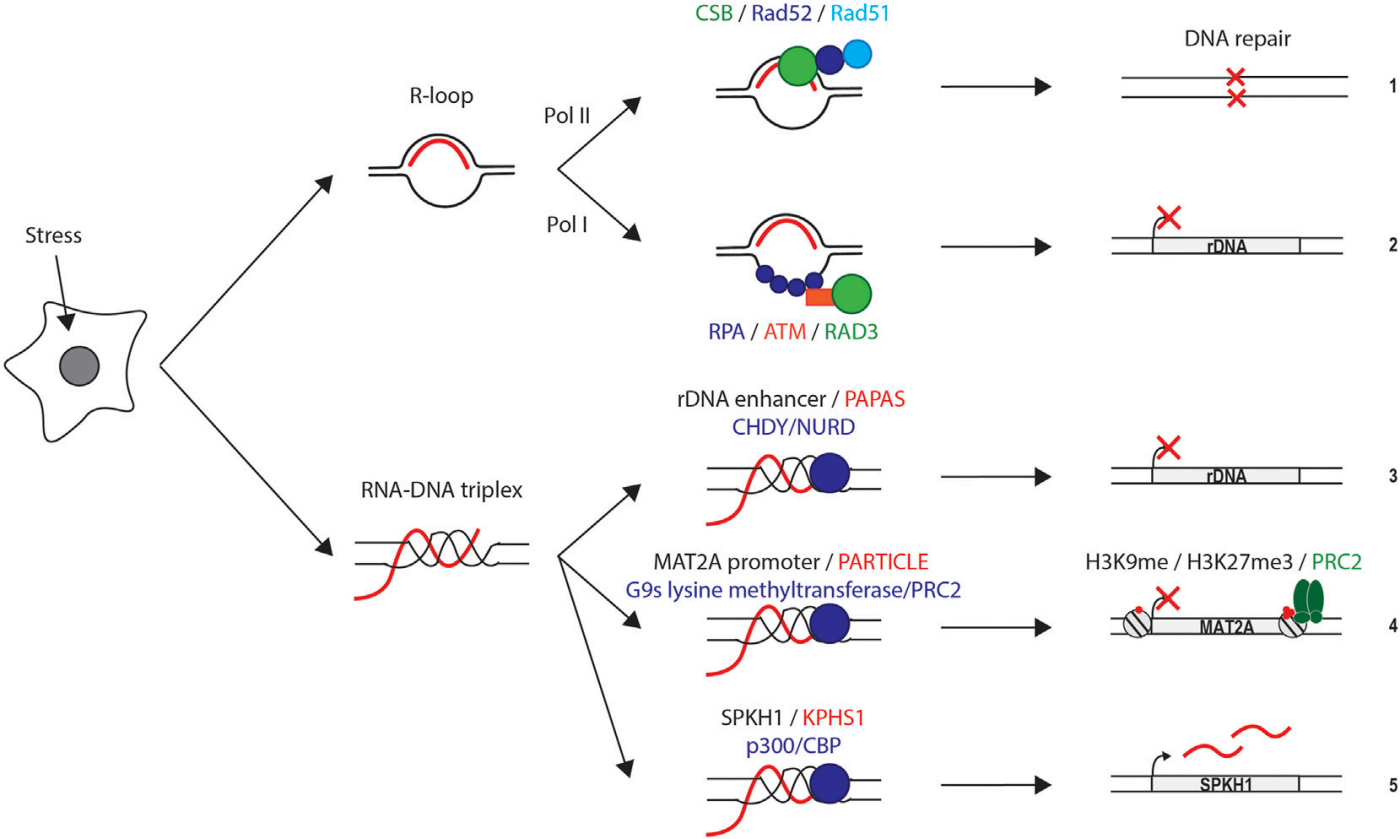
Summary of the moleculecular mechanisms and role of R-loops and DNA:RNA triplexes during stress conditions. Schemes indicate the formation of R-loops and DNA:RNA triplexes, protein recruited to the chromatine, and implication in DNA repair and the expression of protein-encoding and ribosal genes. **(1)** Oxidative stress and reactive oxygen species lead to R-loop formation in transcriptionally active region, which can protect from DNA damage by recruiting DNA damage repair proteins CSB, Rad51, and Rad52. **(2)** Genotoxic stress stabilizes the formation of R-loops over rDNA genes. The displaced ssDNA is bound by RPA leading to the recruitment of ATM and Rad53 and inducing the silencing of rDNA genes. **(3)** Elevated temperatures induced PAPAS transcription which form DNA:RNA triplex over rDNA enhancer. The triplex recruits CHD4 and NuRD which promotes rDNA genes repression. **(4)** Ionizing radiations induce PARTICLE expression which form DNA:RNA triplex in the promoter region of MAT2A gene leading to the recruitment of methyltransferases which repress MAT2A. **(5)** Antisense ncRNA Kphs1 form an DNA:RNA triplex over SPHK1 promoter, recruiting p300/CBP and promoting histone acetylation and SPHK1 transcription.

Like development, stress conditions represent a challenge to the cell because it has to adjust several gene expression pathways simultaneously. A common hallmark of stress conditions is the general shutdown of transcription of ubiquitously expressed protein-encoding and ribosomal biogenesis-related genes (Vihervaara et al., 2018). This response enables cells to decrease the load of damaged molecules downstream of the gene expression pathway and provides time to repair DNA damage. Many of the protein players and molecular mechanisms orchestrating transcription and DNA repair have been well studied. It is also well established that several stresses induce the transcription of diverse ncRNAs in different organisms (McKechnie et al., 1998; Jolly et al., 2004; Audas et al., 2012; Place and Noonan 2014; Goenka et al., 2016; Koskas et al., 2017; Schreiner et al., 2019). However, their mechanism of action and relevance for cell survival to stress have not been yet established. In the last few years, increasing examples suggest that the mode of actions of these ncRNAs is through the formation of non-canonical nucleic acid structures. Hence, stress-related ncRNAs might orchestrate the recruitment of factors to specific transcriptional active regions of the genome to modulate transcription and favor DNA repair (Fig. 6).

### 5.1 R-Loops

There are two recent examples supporting the role of stress-induced R-loop formation in protecting DNA from damage in mammalian cells. Oxidative stress increases the concentration of Reactive Oxygen Species (ROS) that damages the DNA. Hence, ROS can cause mutations that are deleterious for the cell when affecting protein-encoding and ribosomal genes. It has been recently shown that ROS induces the formation of R-loops in transcriptionally active regions of the mammalian genome. Their formation protects DNA from ROS-induced damage by a mechanism that involves transcription-coupled homologous recombination (TC-HR). R-loops recruit other proteins than the canonical BRC1 and BRC2 to the site of DNA damage in order to mediate TC-HR. The RNA-DNA hybrid part of the R-loop is recognized by the Cockayne Syndrome Protein B (CSB) that targets Rad52 to the damaged-transcribed region. Rad52 recruits the recombinase Rad51 that assists in repairing DNA double-strand breaks. (Schieber and Chandel 2014). The relationship between R-loops, Rad52, and DNA break repair has been previously reported in the yeasts *Saccharomyces cerevisiae* and *Schizosaccharomyces pombe* when a double-strand break was induced (Keskin et al., 2014; Ohle et al., 2016; Mazina et al., 2017)**.** Genotoxic stresses also induce transcriptional silencing of ribosomal genes (rDNAs) in the nucleolus. The stabilization of R-loops on rDNAs during hyperosmotic stress conditions leads to a functional cascade of events that repressed Pol-I-dependent transcription. In this case, the ssDNA displaced by the R-loop is coated by the Replication Protein A (RPA) that leads to the recruitment of the ATM- and Rad3-related (ATR) kinase. Once the ATR kinase is activated, it shuts down the transcription of rRNAs (Velichko et al., 2019). Hence, stress-induced R-loops use their RNA-DNA hybrid or the displaced ssDNA to target DNA repair proteins and transcriptional repressors to actively transcribed genes in order to protect the cell from stress-induced damage.

Organismal consequences of R-loop formation in the response to DNA damage have been recently published (Chatzidoukaki et al., 2021). One of the consequences of persistent DNA damage is premature aging. Using a knockout *ERCC* mouse model deficient for DNA repair, Chatzidoukaki and others demonstrated that pancreatic cells of these animals accumulated ssDNA in the cytoplasm, which activates a viral-like immune response. The accumulation of cytoplasmic ssDNA depends on R-loop formation in response to DNA damage. Co-transcriptional formed R-loops cannot be solved because they do not bind the nuclease XPF efficiently in the absence of ERCC. Therefore, DNA damage leads to the accumulation of ssDNA in the nucleus that is further released to the cytoplasm (Chatzidoukaki et al., 2021). The position and sequence of the R-loops detected in this study remain unknown since most of the experiments done to detect them rely on immunofluorescence using the S9.6 antibody.

Localization of R-loops over the genome and its changes in response to environmental stimuli in *Arabidopsis Thaliana* have recently provided an overview of their landscape and dynamics (Xu et al., 2020). In general, R-loops remain stable to changes in environmental conditions except at 84 h of recovery from a long heat shock. Both heat shock and recovery change the patterns and levels of R-loop localized in gene bodies, and antisense R-loops, usually found at the promoter of genes, also increased upon heat shock and recovery. These results suggest that R-loops might regulate the responses to changes in temperature. However, genome-wide changes in R-loop did not reflect changes in RNA levels during recovery from stress. Hence, either R-loops have a limited role during stress or longer recovery, and recurrent stress should be considered to analyze the biological relevance of stress-induced R-loops.

### 5.2 DNA:RNA Triplexes

Similar to R-loops, we have just started discovering that ncRNAs also participate in the stress response by forming DNA:RNA triplexes. Three examples of triplex-forming ncRNAs, *PAPAS*, *PARTICLE,* and *KHPS1*, have been described to regulate gene expression activities during stress conditions (Greifenstein et al., 2021). Elevated temperatures (42°C) upregulate the ncRNA PAPAS transcription (promoter and pre-rRNA antisense) in mammalian cells. Transcription of >10 kb *PAPAS* occurs in an antisense orientation to pre-rRNAs, which favors the *cis*-interaction of *PAPAS* with different regions of the rDNA and the formation of an DNA:RNA triplex at the enhancer of rDNA. The DNA:RNA triplex structure mediates the repression of rDNA transcription through the recruitment of the CHD4 subunit of the nucleosome remodeling and deacetylation NuRD complex to the rDNA promoter. Stress-induced dephosphorylation of CHD4 favors its interaction with the A-rich region of *PAPAs*, further promoting the targeting of chromatin remodeling complexes like the histone methyltransferase Suv4-20h2 to repress the transcription of ribosomal genes upon stress (Zhao et al., 2018). The recruitment of CHD4/NuRD to rDNA by *PAPAS* during heat and hypoosmotic stress also represses transcription by shifting the promoter-bound nucleosomes. Targeting of the adenosine triphosphatase subunit of NuRD leads to the deacetylation of histones and movement of the promoter-bound nucleosome into a position that is refractory to transcription initiation (Zhao et al., 2016b; Zhao et al., 2016a).

Ionizing radiation also represents stress for the cells because it causes macromolecular damage. Low doses of ionizing radiation induce the expression of the ncRNA *PARTICLE* (promoter of MAT2A-antisense radiation-induced circulating ncRNA) in mammalian cells in culture. *PARTICLE* forms a DNA:RNA triplex within the CpG island localized in the promoter ofthe *MAT2A* gene (methionine adenosyltransferase) and suppresses its expression (O’Leary et al., 2015). *PARTICLE* also interacts with the G9s lysine methyltransferase and the SUZ12 subunit of the PRC2 complex targeting the methylation of H3K9 and trimethylation of H3K27 to the MAT2A promoter, respectively, which also mediates its transcriptional silencing (O’Leary et al., 2015). In contrast with these repressive transcriptional functions of *PAPAS* and *PARTICLE*, the human ncRNA *Khps1* favors the transcription of the SPHK1 proto-oncogene to protect cells from apoptosis. The transcription factor E2F1, which plays a role in cell-cycle progression, DNA-damage response, and apoptosis, induces the transcription of *Kphs1* in the antisense orientation of *SPHK1* gene. *Kphs1* forms an DNA:RNA triplex in the *SPHK1* promoter that recruits the histone acetyltransferase p300/CBP. Acetylation of histones favors the open of chromatin and the activation of *SPHK1* transcription by E2F1, which offsets E2F1 proapoptotic activity (Postepska-Igielska et al., 2015).

Overall, the specificity provided by the nucleotide sequence of ncRNAs and their capacity to form DNA:RNA triplexes provides the means to target chromatin remodelers to specific genes and regulate their transcriptional status.

## Discussion

R-loops and DNA:RNA triplexes involving coding and ncRNA are present throughout the genome of various organisms ranging from yeast to humans. As presented in this review, these non-canonical nucleic acid structures involving ncRNA participate in the transcriptional activation and repression of protein-coding genes by targeting transcription factors or epigenetic and DNA modifiers lacking sequence-specific DNA binding activity. We have highlighted examples that provide detailed information about the molecular mechanisms underlying gene expression regulation. However, few mechanistic examples have been the object of in-depth analysis. Non-canonical nucleic acids might rather provide a structure instead of a sequence to be recognized by a protein. Therefore, these structures may constitute an additional general regulator of gene expression, which might be essential for development and survival to stress.

To this day, all the techniques available to study non-canonical nucleic acid structures have limitations. The common caveat to all of them is their lack of capacity to identify the RNA and the DNA part simultaneously. In mammals (Sanz et al., 2016), *Arabidopsis thaliana* (Xu et al., 2017)*,* and *Drosophila melanogaster* (Alecki et al., 2020)*,* 8 to 15% of the R-loops are formed at intergenic regions. In *Drosophila melanogaster*, 20% of the R-loops don’t overlap with any annotated transcript. Hence, R-loops can be formed in trans between DNA and RNA with potentially microhomology regions of ten to thirty nucleotides. Developing specific antibodies or markers to identify DNA:RNA triplexes *in vivo* and improving the specificity of S9.6 for imaging purposes (Smolka et al., 2021) will allow the identification of these structures within individual cells. Consequently, the number of RNAs that can act *in trans* to form non-canonical nucleic acid structures might be underestimated. Although few studies have looked at RNA-DNA hybrid, the main part of an R-loop, to identify its interacting proteins by mass spectrometry, no attention has been given to the remaining part of the R-loop, the dsDNA-ssDNA-RNA-DNA hybrid junction, nor the DNA:RNA triplex. Additionally, these techniques lack the temporal resolution needed to study the dynamics of formation and resolution of R-loops in the context of the cell and the organisms during development.

Proteins responsible for the resolution of R-loops have been identified, but it is still unknown which proteins participate in the formation or resolution of DNA:RNA triplexes. The main R-loop resolving proteins are RNase H1 and H2, RNA-DNA helicases, and topoisomerases (Hyjek et al., 2019; Chon et al., 2009; Kim et al., 1999; Sollier et al., 2014; Chakraborty and Grosse 2011; Manzo et al., 2018; Zhang et al., 2019). The knockdown or overexpression of RNase H1 has limited and controversial effects on R-loops genome-wide, and RNase H2 and RNA-DNA helicases affect many R-loops, which makes it difficult to conclude on a general function of R-loops because these approaches stress cells. Topoisomerases cannot be knockdown in higher eukaryotes because they are essential as they reduce the level of supercoiling DNA during DNA replication and transcription (Lee et al., 1993; Morham et al., 1996; McClendon et al., 2005; Plank et al., 2005; Baranello et al., 2016; Pommier et al., 2016; Capranico et al., 2017; Achar et al., 2020). Hence, their role on R-loop resolution has not been studied in higher eukaryotes. Our understanding of the function of specific R-loop in gene regulation requires tools to remove specific R-loops without affecting the remaining others instead of removing the RNA and the DNA that composed them. These approaches complemented with high-resolution imaging will provide the spatiotemporal resolution required to study their nuclear localization and dynamics over development and stress (Sato et al., 2020). We foresee that these technologies will widen our knowledge on their formation and resolution and the capacity to investigate their specific functions in higher organisms.

## References

[B1] Achar Y. J. Adhil M. Choudhary R. Gilbert N. Foiani M. Foiani Marco. (2020). Negative Supercoil at Gene Boundaries Modulates Gene Topology. Nature 577 (7792), 701–705. 10.1038/s41586-020-1934-4 31969709

[B2] Alecki C. Francis N. J. (2021). Identification of R-Loop-Forming Sequences in Drosophila Melanogaster Embryos and Tissue Culture Cells Using DRIP-Seq. Bio Protoc. 11 (9), e4011. 10.21769/BioProtoc.4011 PMC8161117 34124311

[B3] Alecki C. Chiwara V. Sanz L. A. Grau D. Arias Pérez O. Boulier E. L. (2020). RNA-DNA Strand Exchange by the Drosophila Polycomb Complex PRC2. Nat. Commun. 11 (1), 1–14. 10.1038/s41467-020-15609-x 32286294 PMC7156742

[B4] An B. Kameda T. Imamura T. (2021). The Evolutionary Acquisition and Mode of Functions of Promoter-Associated Non-coding RNAs (PancRNAs) for Mammalian Development. Essays Biochem. 65 (4), 697–708. 10.1042/EBC20200143 34328174

[B5] Arab K. Karaulanov E. Musheev M. Trnka P. Schäfer A. Grummt I. (2019). GADD45A Binds R-Loops and Recruits TET1 to CpG Island Promoters. Nat. Genet. 51 (2), 217–223. 10.1038/s41588-018-0306-6 30617255 PMC6420098

[B6] Audas T. E. Jacob M. D. Lee S. (2012). Immobilization of Proteins in the Nucleolus by Ribosomal Intergenic Spacer Noncoding RNA. Mol. Cel 45 (2), 147–157. 10.1016/j.molcel.2011.12.012 22284675

[B7] Bacolla A. Wang G. Vasquez K. M. (2015). New Perspectives on DNA and RNA Triplexes as Effectors of Biological Activity. Plos Genet. 11 (12), e1005696. 10.1371/journal.pgen.1005696 26700634 PMC4689454

[B8] Balas Maggie. M. Hartwick Erik. W. Barrington Chloe. Roberts Justin. T. Wu Stephen. K. Ryan Bettcher. (2020). RNA Matchmaking Remodels LncRNA Structure and Promotes PRC2 Activity. April, 2020.04.13.040071. BioRxiv. 10.1101/2020.04.13.040071 PMC8046370 33853770

[B9] Baranello L. Wojtowicz D. Cui K. Devaiah B. N. Chung H.-J. Chan-Salis K. Y. (2016). RNA Polymerase II Regulates Topoisomerase 1 Activity to Favor Efficient Transcription. Cell 165 (2), 357–371. 10.1016/j.cell.2016.02.036 27058666 PMC4826470

[B10] Battich N. Stoeger T. Pelkmans L. (2013). Image-Based Transcriptomics in Thousands of Single Human Cells at Single-Molecule Resolution. Nat. Methods 10 (11), 1127–1133. 10.1038/nmeth.2657 24097269

[B134] Benitez-Guijarro M. Lopez-Ruiz C. Tarnauskaite Z. Murina O. Mohammad M. M. Williams T. C. (2018). RNase H_2_, Mutated in Aicardi-Goutieres Syndrome, Promotes LINE-1 Retrotransposition. EMBO J. 37 (15), e98506. 10.15252/embj.201798506 29959219 PMC6068448

[B11] Cabili M. N. Dunagin M. C. McClanahan P. D. Biaesch A. Padovan-Merhar O. Regev A. (2015). Localization and Abundance Analysis of Human LncRNAs at Single-Cell and Single-Molecule Resolution. Genome Biol. 16 (1), 20. 10.1186/s13059-015-0586-4 25630241 PMC4369099

[B12] Capranico G. Marinello J. Chillemi G. (2017). Type I DNA Topoisomerases. J. Med. Chem. 60 (6), 2169–2192. 10.1021/acs.jmedchem.6b00966 28072526

[B13] Cargill M. Venkataraman R. Lee S. (2021). DEAD-box RNA Helicases and Genome Stability. Genes 12 (10), 1471. 10.3390/genes12101471 34680866 PMC8535883

[B14] Chakraborty P. Grosse F. (2011). Human DHX9 Helicase Preferentially Unwinds RNA-Containing Displacement Loops (R-Loops) and G-Quadruplexes. DNA Repair 10 (6), 654–665. 10.1016/j.dnarep.2011.04.013 21561811

[B15] Chatzidoukaki O. Stratigi K. Goulielmaki E. Niotis G. Akalestou-Clocher A. Gkirtzimanaki K. (2021). R-loops Trigger the Release of Cytoplasmic SsDNAs Leading to Chronic Inflammation upon DNA Damage. Sci. Adv. 7 (47), eabj5769. 10.1126/sciadv.abj5769 34797720 PMC8604417

[B16] Chellini L. Frezza V. Paronetto M. P. (2020). Dissecting the Transcriptional Regulatory Networks of Promoter-Associated Noncoding RNAs in Development and Cancer. J. Exp. Clin. Cancer Res. 39 (March), 51. 10.1186/s13046-020-01552-8 32183847 PMC7079525

[B135] Chen L. Chen J.-Y. Zhang X. Ying G. Xiao R. Shao C. (2017). R-ChIP Using Inactive RNase H Reveals Dynamic Coupling of R-loops with Transcriptional Pausing at Gene Promoters. Mol. Cell. 68 (4), 745–757.e5. 10.1016/j.molcel.2017.10.008 29104020 PMC5957070

[B136] Chen J.-Y. Zhang X. Fu X.-D. Chen L. (2019). R-ChIP for Genome-Wide Mapping of R-Loops by Using Catalytically Inactive RNASEH1. Nat Protoc. 14 (5), 1661–1685. 10.1038/s41596-019-0154-6 30996261 PMC6604627

[B17] Chon H. Vassilev A. DePamphilis M. L. Zhao Y. Zhang J. Burgers P. M. (2009). Contributions of the Two Accessory Subunits, RNASEH2B and RNASEH2C, to the Activity and Properties of the Human RNase H2 Complex. Nucleic Acids Res. 37 (1), 96–110. 10.1093/nar/gkn913 19015152 PMC2615623

[B18] Costa S. Dean C. (2019). Storing Memories: The Distinct Phases of Polycomb-Mediated Silencing of Arabidopsis FLC. Biochem. Soc. Trans. 47 (4), 1187–1196. 10.1042/BST20190255 31278155

[B19] Crevillén P. Sonmez C. Wu Z. Dean C. (2013). A Gene Loop Containing the Floral Repressor FLC Is Disrupted in the Early Phase of Vernalization. Embo J. 32 (1), 140–148. 10.1038/emboj.2012.324 23222483 PMC3545306

[B20] Cristini A. Groh M. Kristiansen M. S. Gromak N. (2018). RNA/DNA Hybrid Interactome Identifies DXH9 as a Molecular Player in Transcriptional Termination and R-Loop-Associated DNA Damage. Cel Rep. 23 (6), 1891–1905. 10.1016/j.celrep.2018.04.025 PMC5976580 29742442

[B21] Crossley M. P. Bocek M. Cimprich K. A. (2019). R-loops as Cellular Regulators and Genomic Threats. Mol. Cel 73 (3), 398–411. 10.1016/j.molcel.2019.01.024 PMC6402819 30735654

[B22] Cusanelli E. Romero C. A. P. Chartrand P. (2013). Telomeric Noncoding RNA TERRA Is Induced by Telomere Shortening to Nucleate Telomerase Molecules at Short Telomeres. Mol. Cel 51 (6), 780–791. 10.1016/j.molcel.2013.08.029 24074956

[B23] Derrien T. Johnson R. Bussotti G. Tanzer A. Djebali S. Tilgner H. (2012). The GENCODE V7 Catalog of Human Long Noncoding RNAs: Analysis of Their Gene Structure, Evolution, and Expression. Genome Res. 22 (9), 1775–1789. 10.1101/gr.132159.111 22955988 PMC3431493

[B24] Fang Y. Chen L. Lin K. Feng Y. Zhang P. Pan X. (2019). Characterization of Functional Relationships of R-Loops with Gene Transcription and Epigenetic Modifications in rice. Genome Res. 29, 1287–1297. gr.246009. 10.1101/gr.246009.118 31262943 PMC6673715

[B25] Femino A. M. Fay F. S. Fogarty K. Singer R. H. (1998). Visualization of Single RNA Transcripts *In Situ* . Science 280 (5363), 585–590. 10.1126/science.280.5363.585 9554849

[B26] Fernandes J. Acuña S. Juliana I. Aoki Lucile. Floeter-Winter M. Aoki J. (2019). Long Non-coding RNAs in the Regulation of Gene Expression: Physiology and Disease. ncRNA 5 (1), 17. 10.3390/ncrna5010017 PMC6468922 30781588

[B27] Gilbert Scott. F. (2000). The Origins of Anterior-Posterior PolarityDevelopmental Biology. 6th Edition. Available at: https://www.ncbi.nlm.nih.gov/books/NBK10039/ .

[B28] Ginno P. A. Lott P. L. Christensen H. C. Korf I. Chédin F. (2012). R-loop Formation Is a Distinctive Characteristic of Unmethylated Human CpG Island Promoters. Mol. Cel 45 (6), 814–825. 10.1016/j.molcel.2012.01.017 PMC3319272 22387027

[B29] Goenka A. Sengupta S. Pandey R. Parihar R. Mohanta G. C. Mukerji M. (2016). Human Satellite-III Non-coding RNAs Modulate Heat Shock-Induced Transcriptional Repression. J. Cel Sci. 129 (19), 3541–3552. 10.1242/jcs.189803 27528402

[B30] Gong J. Ju Y. Shao D. Zhang Q. C. (2018). Advances and Challenges towards the Study of RNA-RNA Interactions in a Transcriptome-wide Scale. Quant Biol. 6 (3), 239–252. 10.1007/s40484-018-0146-5

[B31] Greifenstein A. A. Jo S. Bierhoff H. (2021). RNA:DNA Triple Helices: from peculiar Structures to Pervasive Chromatin Regulators. Essays Biochem. 65 (4), 731–740. 10.1042/EBC20200089 33835128

[B32] Grote P. Herrmann B. G. (2013). The Long Non-coding RNAFendrrlinks Epigenetic Control Mechanisms to Gene Regulatory Networks in Mammalian Embryogenesis. RNA Biol. 10 (10), 1579–1585. 10.4161/rna.26165 24036695 PMC3866236

[B33] Grote P. Wittler L. Hendrix D. Koch F. Währisch S. Beisaw A. (2013). The Tissue-specific LncRNA Fendrr Is an Essential Regulator of Heart and Body Wall Development in the Mouse. Dev. Cel 24 (2), 206–214. 10.1016/j.devcel.2012.12.012 PMC4149175 23369715

[B34] Guetg C. Lienemann P. Sirri V. Grummt I. Hernandez-Verdun D. Hottiger M. O. (2010). The NoRC Complex Mediates the Heterochromatin Formation and Stability of Silent RRNA Genes and Centromeric Repeats. Embo J. 29 (13), 2135–2146. 10.1038/emboj.2010.17 20168299 PMC2905252

[B35] Gupta R. A. Shah N. Wang K. C. Kim J. Wong David. J. Horlings H. M. (2010). Long Non-coding RNA HOTAIR Reprograms Chromatin State to Promote Cancer Metastasis. Nature 464 (7291), 1071–1076. 10.1038/nature08975 20393566 PMC3049919

[B36] Hafner M. Katsantoni M. Köster T. Marks J. Mukherjee J. Staiger D. (2021). CLIP and Complementary Methods. Nat. Rev. Methods Primers 1 (1), 1–23. 10.1038/s43586-021-00018-1

[B37] Hajirnis N. Mishra R. K. (2021). Homeotic Genes: Clustering, Modularity, and Diversity. Front. Cel Dev. Biol. 9 (August), 718308. 10.3389/fcell.2021.718308 PMC8386295 34458272

[B38] Hamazaki N. Uesaka M. Nakashima K. Agata K. Imamura T. (2015). Gene Activation-Associated Long Noncoding RNAs Function in Mouse Preimplantation Development. Development (Cambridge, England) 142 (5), 910–920. 10.1242/dev.116996 PMC4352986 25633350

[B39] Hartono S. R. Malapert A. Legros P. Bernard P. Chédin F. Vanoosthuyse V. (2018). Amélie Malapert, Pénélope Legros, Pascal Bernard, Frédéric Chédin, and Vincent VanoosthuyseThe Affinity of the S9.6 Antibody for Double-Stranded RNAs Impacts the Accurate Mapping of R-Loops in Fission Yeast. J. Mol. Biol. 430 (3), 272–284. 10.1016/j.jmb.2017.12.016 29289567 PMC5987549

[B40] Hyjek M. Figiel M. Nowotny M. (2019). RNases H: Structure and Mechanism. DNA Repair 84 (December), 102672. 10.1016/j.dnarep.2019.102672 31371183

[B41] Jolly C. Metz A. Govin J. Vigneron M. Turner B. M. Khochbin S. (2004). Stress-Induced Transcription of Satellite III Repeats. J. Cel Biol. 164 (1), 25–33. 10.1083/jcb.200306104 PMC2171959 14699086

[B42] Kaneko S. Son J. Shen S. S. Reinberg D. Bonasio R. (2013). PRC2 Binds Active Promoters and Contacts Nascent RNAs in Embryonic Stem Cells. Nat. Struct. Mol. Biol. 20 (11), 1258–1264. 10.1038/nsmb.2700 24141703 PMC3839660

[B43] Keskin H. Shen Y. Huang F. Patel M. Yang T. Ashley K. (2014). Transcript-RNA-templated DNA Recombination and Repair. Nature 515 (7527), 436–439. 10.1038/nature13682 25186730 PMC4899968

[B44] Khalil A. M. Guttman M. Huarte M. Garber M. Raj A. Rivea Morales D. (2009). Many Human Large Intergenic Noncoding RNAs Associate with Chromatin-Modifying Complexes and Affect Gene Expression. Pnas 106 (28), 11667–11672. 10.1073/pnas.0904715106 19571010 PMC2704857

[B45] Kim D.-H. Sung S. (2017). Vernalization-Triggered Intragenic Chromatin Loop Formation by Long Noncoding RNAs. Dev. Cel 40, 302–312. 10.1016/j.devcel.2016.12.021 PMC5303624 28132848

[B46] Kim D.-H. Xi Y. Sung S. (2017). Modular Function of Long Noncoding RNA, COLDAIR, in the Vernalization Response. Plos Genet. 13 (7), e1006939. 10.1371/journal.pgen.1006939 28759577 PMC5552341

[B47] Kim H.-D. Choe J. Seo Y.-S. (1999). The Sen1+ Gene of Schizosaccharomyces Pombe, a Homologue of Budding Yeast SEN1, Encodes an RNA and DNA Helicase. Biochemistry 38 (44), 14697–14710. 10.1021/bi991470c 10545196

[B48] König F. Schubert T. Längst G. (2017). The Monoclonal S9.6 Antibody Exhibits Highly Variable Binding Affinities towards Different R-Loop Sequences. PLOS ONE 12 (6), e0178875. 10.1371/journal.pone.0178875 28594954 PMC5464589

[B49] Koskas S. Decottignies A. Dufour S. Pezet M. Verdel A. Vourc’h C. (2017). Heat Shock Factor 1 Promotes TERRA Transcription and Telomere Protection upon Heat Stress. Nucleic Acids Res. 45 (11), 6321–6333. 10.1093/nar/gkx208 28369628 PMC5499866

[B50] Kwon S. (2013). Single-Molecule Fluorescence *In Situ* Hybridization: Quantitative Imaging of Single RNA Molecules. BMB Rep. 46 (2), 65–72. 10.5483/BMBRep.2013.46.2.016 23433107 PMC4133856

[B51] Lee M. P. Brown S. D. Chen A. Hsieh T. S. (1993). DNA Topoisomerase I Is Essential in Drosophila Melanogaster. Proc. Natl. Acad. Sci. 90 (14), 6656–6660. 10.1073/pnas.90.14.6656 8393572 PMC46991

[B52] Lenstra T. L. Coulon A. Chow C. C. Larson D. R. (2015). Single-Molecule Imaging Reveals a Switch between Spurious and Functional NcRNA Transcription. Mol. Cel 60 (4), 597–610. 10.1016/j.molcel.2015.09.028 PMC4656089 26549684

[B53] Li L. Liu B. Wapinski O. L. Tsai M.-C. Qu K. Zhang J. (2013). Targeted Disruption of Hotair Leads to Homeotic Transformation and Gene Derepression. Cel Rep. 5 (1), 3–12. 10.1016/j.celrep.2013.09.003 PMC4038295 24075995

[B54] Li Y. Song Y. Xu W. Li Q. Wang X. Li K. (2020). R-loops Coordinate with SOX2 in Regulating Reprogramming to Pluripotency. Sci. Adv. 6 (24), eaba0777. 10.1126/sciadv.aba0777 32704541 PMC7360481

[B55] Li Y. Syed J. Sugiyama H. (2016). RNA-DNA Triplex Formation by Long Noncoding RNAs. Cel Chem. Biol. 23 (11), 1325–1333. 10.1016/j.chembiol.2016.09.011 27773629

[B56] Lim Y. W. Sanz L. A. Xu X. Hartono S. R. Chédin F. (2015). Genome-wide DNA Hypomethylation and RNA:DNA Hybrid Accumulation in Aicardi-Goutières Syndrome. ELife 4. 10.7554/eLife.08007 PMC4528086 26182405

[B57] Lin C. Miles W. O. (2019). Beyond CLIP: Advances and Opportunities to Measure RBP-RNA and RNA-RNA Interactions. Nucleic Acids Res. 47 (11), 5490–5501. 10.1093/nar/gkz295 31076772 PMC6582316

[B58] Lockhart A. Pires V. B. Bento F. Kellner V. Luke-Glaser S. Yakoub G. (2019). RNase H1 and H2 Are Differentially Regulated to Process RNA-DNA Hybrids. Cel Rep. 29 (9), 2890–2900.e5. 10.1016/j.celrep.2019.10.108 31775053

[B59] Lotfi C. F. P. Passaia B. S. Kremer J. L. (2021). Role of the BHLH Transcription Factor TCF21 in Development and Tumorigenesis. Braz. J. Med. Biol. Res. 54 (5), e10637. 10.1590/1414-431X202010637 33729392 PMC7959166

[B60] Lu Z. Chang H. Y. (2018). The RNA Base-Pairing Problem and Base-Pairing Solutions. Cold Spring Harb Perspect. Biol. 10 (12), a034926. 10.1101/cshperspect.a034926 30510063 PMC6280703

[B61] Malig M. Chedin F. (2020). Characterization of R-Loop Structures Using Single-Molecule R-Loop Footprinting and Sequencing. Methods Mol. Biol. (Clifton, N.J.) 2161, 209–228. 10.1007/978-1-0716-0680-3_15 PMC7669279 32681515

[B62] Mallo M. Wellik D. M. Deschamps J. (2010). Hox Genes and Regional Patterning of the Vertebrate Body Plan. Dev. Biol. 344 (1), 7–15. 10.1016/j.ydbio.2010.04.024 20435029 PMC2909379

[B63] Manzo S. G. Hartono S. R. Sanz L. A. Marinello J. De Biasi S. Cossarizza A. (2018). DNA Topoisomerase I Differentially Modulates R-Loops across the Human Genome. Genome Biol. 19 (July). 10.1186/s13059-018-1478-1 PMC6066927 30060749

[B64] Mayer C. Neubert M. Grummt I. (2008). The Structure of NoRC‐associated RNA Is Crucial for Targeting the Chromatin Remodelling Complex NoRC to the Nucleolus. EMBO Rep. 9 (8), 774–780. 10.1038/embor.2008.109 18600236 PMC2515205

[B65] Mazina O. M. Keskin H. Hanamshet K. Storici F. Mazin A. V. (2017). Rad52 Inverse Strand Exchange Drives RNA-Templated DNA Double-Strand Break Repair. Mol. Cel 67 (1), 19–29.e3. 10.1016/j.molcel.2017.05.019 PMC5547995 28602639

[B66] McClendon A. K. Rodriguez A. C. Osheroff N. (2005). Human Topoisomerase IIα Rapidly Relaxes Positively Supercoiled DNA. J. Biol. Chem. 280 (47), 39337–39345. 10.1074/jbc.M503320200 16188892

[B67] McKechnie S. W. Halford M. M. McColl G. Hoffmann A. A. (1998). Both Allelic Variation and Expression of Nuclear and Cytoplasmic Transcripts of Hsr-Omega Are Closely Associated with Thermal Phenotype in Drosophila. Proc. Natl. Acad. Sci. 95 (5), 2423–2428. 10.1073/pnas.95.5.2423 9482901 PMC19362

[B68] Miglietta G. Russo M. Capranico G. (2020). G-quadruplex-R-loop Interactions and the Mechanism of Anticancer G-Quadruplex Binders. Nucleic Acids Res. 48 (21), 11942–11957. 10.1093/nar/gkaa944 33137181 PMC7708042

[B69] Mondal T. Subhash S. Vaid R. Enroth S. Uday S. Reinius B. (2015). MEG3 Long Noncoding RNA Regulates the TGF-β Pathway Genes through Formation of RNA-DNA Triplex Structures. Nat. Commun. 6 (1), 7743. 10.1038/ncomms8743 26205790 PMC4525211

[B70] Morgan A. R. Wells R. D. (1968). Specificity of the Three-Stranded Complex Formation between Double-Stranded DNA and Single-Stranded RNA Containing Repeating Nucleotide Sequences. J. Mol. Biol. 37 (1), 63–80. 10.1016/0022-2836(68)90073-9 5760495

[B71] Morham S. G. Kluckman K. D. Voulomanos N. Smithies O. (1996). Targeted Disruption of the Mouse Topoisomerase I Gene by Camptothecin Selection. Mol. Cel Biol 16 (12), 6804–6809. 10.1128/MCB.16.12.6804 PMC231683 8943335

[B72] Moye A. L. Porter K. C. Cohen Scott. B. Phan Tram. Cohen S. B. Phan T. (2015). Telomeric G-Quadruplexes Are a Substrate and Site of Localization for Human Telomerase. Nat. Commun. 6 (July). 10.1038/ncomms8643 PMC4510649 26158869

[B73] Mueller F. Senecal A. Tantale K. Marie-Nelly H. Ly N. Collin O. (2013). FISH-quant: Automatic Counting of Transcripts in 3D FISH Images. Nat. Methods 10 (4), 277–278. 10.1038/nmeth.2406 23538861

[B74] Niehrs C. Luke B. (2020). Regulatory R-Loops as Facilitators of Gene Expression and Genome Stability. Nat. Rev. Mol. Cel Biol 21, 167–178. 10.1038/s41580-019-0206-3 PMC7116639 32005969

[B75] Nowotny M. Cerritelli S. M. Ghirlando R. Gaidamakov S. A. Crouch R. J. Yang W. (2008). Specific Recognition of RNA/DNA Hybrid and Enhancement of Human RNase H1 Activity by HBD. Embo J. 27 (7), 1172–1181. 10.1038/emboj.2008.44 18337749 PMC2323259

[B76] O'Brien J. Hayder H. Zayed Y. Peng C. (2018). Overview of MicroRNA Biogenesis, Mechanisms of Actions, and Circulation. Front. Endocrinol. 9, 402. 10.3389/fendo.2018.00402 PMC6085463 30123182

[B77] Ohle C. Tesorero R. Schermann G. Dobrev N. Sinning I. Fischer T. (2016). Transient RNA-DNA Hybrids Are Required for Efficient Double-Strand Break Repair. Cell 167 (4), 1001–1013.e7. 10.1016/j.cell.2016.10.001 27881299

[B78] O’Leary V. B. Ovsepian S. V. Carrascosa L. G. Buske F. A. Radulovic V. Niyazi M. (2015). PARTICLE, a Triplex-Forming Long NcRNA, Regulates Locus-specific Methylation in Response to Low-Dose Irradiation. Cel Rep. 11 (3), 474–485. 10.1016/j.celrep.2015.03.043 25900080

[B79] Phillips D. D. Garboczi D. N. Singh K. Hu Z. Leppla S. H. Leysath C. E. (2013). The Sub-nanomolar Binding of DNA-RNA Hybrids by the Single-Chain Fv Fragment of Antibody S9.6. J. Mol. Recognit. 26 (8), 376–381. 10.1002/jmr.2284 23784994 PMC4061737

[B80] Place R. F. Noonan E. J. (2014). Non-Coding RNAs Turn up the Heat: An Emerging Layer of Novel Regulators in the Mammalian Heat Shock Response. Cell Stress and Chaperones 19 (2), 159–172. 10.1007/s12192-013-0456-5 24002685 PMC3933615

[B81] Plank J. L. Chu S. H. Pohlhaus J. R. Wilson-Sali T. Hsieh T.-s. (2005). *Drosophila melanogaster* Topoisomerase IIIα Preferentially Relaxes a Positively or Negatively Supercoiled Bubble Substrate and Is Essential during Development. J. Biol. Chem. 280 (5), 3564–3573. 10.1074/jbc.M411337200 15537633

[B82] Pommier Y. Sun Y. Huang S.-y. N. Nitiss J. L. (2016). Roles of Eukaryotic Topoisomerases in Transcription, Replication and Genomic Stability. Nat. Rev. Mol. Cel Biol 17 (11), 703–721. 10.1038/nrm.2016.111 PMC9248348 27649880

[B83] Postepska-Igielska A. Giwojna A. Gasri-Plotnitsky L. Schmitt N. Dold A. Ginsberg D. (2015). LncRNA Khps1 Regulates Expression of the Proto-Oncogene SPHK1 via Triplex-Mediated Changes in Chromatin Structure. Mol. Cel 60 (4), 626–636. 10.1016/j.molcel.2015.10.001 26590717

[B84] Radhakrishnan I. Patel D. J. (1994). Solution Structure and Hydration Patterns of a Pyrimidine?Purine?Pyrimidine DNA Triplex Containing a Novel T?CG Base-Triple. J. Mol. Biol. 241 (4), 600–619. 10.1006/jmbi.1994.1534 8057381

[B85] Ramanathan M. Porter D. F. Khavari P. A. (2019). Methods to Study RNA-Protein Interactions. Nat. Methods 16 (3), 225–234. 10.1038/s41592-019-0330-1 30804549 PMC6692137

[B86] Rinn J. L. Kertesz M. Wang J. K. Wang Sharon. Squazzo L. Squazzo S. L. (2007). Functional Demarcation of Active and Silent Chromatin Domains in Human HOX Loci by Noncoding RNAs. Cell 129 (7), 1311–1323. 10.1016/j.cell.2007.05.022 17604720 PMC2084369

[B87] Rosa S. Duncan S. Dean C. (2016). Mutually Exclusive Sense-Antisense Transcription at FLC Facilitates Environmentally Induced Gene Repression. Nat. Commun. 7 (1), 1–7. 10.1038/ncomms13031 PMC5059766 27713408

[B88] Santoro R. Schmitz K. M. Sandoval J. Grummt I. (2010). Intergenic Transcripts Originating from a Subclass of Ribosomal DNA Repeats Silence Ribosomal RNA Genes in *Trans* . EMBO Rep. 11 (1), 52–58. 10.1038/embor.2009.254 20010804 PMC2816622

[B89] Sanz L. A. Chédin. F. (2019). High-resolution, Strand-specific R-Loop Mapping via S9.6-based DNA-RNA Immunoprecipitation and High-Throughput Sequencing. Nat. Protoc. 14 (6), 1734–1755. 10.1038/s41596-019-0159-1 31053798 PMC6615061

[B90] Sanz L. A. Hartono S. R. Lim Y. W. Steyaert S. Rajpurkar A. Ginno P. A. (2016). Prevalent, Dynamic, and Conserved R-Loop Structures Associate with Specific Epigenomic Signatures in Mammals. Mol. Cel 63 (1), 167–178. 10.1016/j.molcel.2016.05.032 PMC4955522 27373332

[B91] Sato H. Das S. Singer R. H. Vera M. (2020). Imaging of DNA and RNA in Living Eukaryotic Cells to Reveal Spatiotemporal Dynamics of Gene Expression. Annu. Rev. Biochem. 89 (1), 159–187. 10.1146/annurev-biochem-011520-104955 32176523 PMC7608990

[B92] Schieber M. Chandel N. S. (2014). ROS Function in Redox Signaling and Oxidative Stress. Curr. Biol. 24 (10), R453–R462. 10.1016/j.cub.2014.03.034 24845678 PMC4055301

[B93] Schmitz K.-M. Mayer C. Postepska A. Grummt I. (2010). Interaction of Noncoding RNA with the RDNA Promoter Mediates Recruitment of DNMT3b and Silencing of RRNA Genes. Genes Dev. 24 (20), 2264–2269. 10.1101/gad.590910 20952535 PMC2956204

[B94] Schreiner W. P. Pagliuso D. C. Garrigues J. M. Chen J. S. Aalto A. P. Pasquinelli A. E. (2019). Remodeling of the Caenorhabditis Elegans Non-coding RNA Transcriptome by Heat Shock. Nucleic Acids Res. 47 (18), 9829–9841. 10.1093/nar/gkz693 31396626 PMC6765114

[B95] Sentürk Cetin N. Kuo C.-C. Ribarska T. Li R. Costa I. G. Grummt I. (2019). Isolation and Genome-wide Characterization of Cellular DNA:RNA Triplex Structures. Nucleic Acids Res. 47 (5), 2306–2321. 10.1093/nar/gky1305 30605520 PMC6411930

[B96] Sinnamon J. R. Czaplinski K. (2014). RNA Detection *In Situ* with FISH-STICs. RNA 20 (2), 260–266. 10.1261/rna.041905.113 24345395 PMC3895277

[B97] Skourti-Stathaki K. Torlai Triglia E. Warburton M. Voigt P. Bird A. Pombo A. (2019). R-loops Enhance Polycomb Repression at a Subset of Developmental Regulator Genes. Mol. Cel 73 (5), 930–945.e4. 10.1016/j.molcel.2018.12.016 PMC6414425 30709709

[B98] Slobodin B. Gerst J. E. (2010). A Novel MRNA Affinity Purification Technique for the Identification of Interacting Proteins and Transcripts in Ribonucleoprotein Complexes. RNA 16 (11), 2277–2290. 10.1261/rna.2091710 20876833 PMC2957065

[B99] Smolka J. A. Sanz L. A. Hartono S. R. Chédin F. (2021). Recognition of RNA by the S9.6 Antibody Creates Pervasive Artifacts when Imaging RNA:DNA Hybrids. J. Cel Biol. 220 (6). 10.1083/jcb.202004079 PMC8040515 33830170

[B100] Sollier J. Stork C. T. García-Rubio M. L. Paulsen R. D. Aguilera A. Cimprich K. A. (2014). Transcription-Coupled Nucleotide Excision Repair Factors Promote R-Loop-Induced Genome Instability. Mol. Cel 56 (6), 777–785. 10.1016/j.molcel.2014.10.020 PMC4272638 25435140

[B101] Sperling A. S. Gibson C. J. Ebert B. L. (2017). The Genetics of Myelodysplastic Syndrome: From Clonal Haematopoiesis to Secondary Leukaemia. Nat. Rev. Cancer 17 (1), 5–19. 10.1038/nrc.2016.112 27834397 PMC5470392

[B137] Statello L. Guo C. J. Chen L. L. Huarte M. (2021). Gene Regulation by Long Non-Coding RNAs and its Biological Functions. Nat. Rev. Mol. Cell Biol. 22, 96–118. 10.1038/s41580-020-00315-9 33353982 PMC7754182

[B102] Steffen P. A. Ringrose. L. (2014). What Are Memories Made of? How Polycomb and Trithorax Proteins Mediate Epigenetic Memory. Nat. Rev. Mol. Cel Biol 15 (5), 340–356. 10.1038/nrm3789 24755934

[B103] Sun Q. Csorba T. Skourti-Stathaki K. Proudfoot N. J. Dean C. (2013). R-loop Stabilization Represses Antisense Transcription at the Arabidopsis FLC Locus. Science 340 (6132), 619–621. 10.1126/science.1234848 23641115 PMC5144995

[B104] Sun Y.-M. Chen Y.-Q. (2020). Principles and Innovative Technologies for Decrypting Noncoding RNAs: From Discovery and Functional Prediction to Clinical Application. J. Hematol. Oncol. 13 (1), 109. 10.1186/s13045-020-00945-8 32778133 PMC7416809

[B105] Tsai M.-C. Manor O. Wan Y. Mosammaparast N. Wang J. K. Lan F. (2010). Long Noncoding RNA as Modular Scaffold of Histone Modification Complexes. Science 329 (5992), 689–693. 10.1126/science.1192002 20616235 PMC2967777

[B106] Uesaka M. Nishimura O. Go Y. Nakashima K. Agata K. Imamura T. (2014). Bidirectional Promoters Are the Major Source of Gene Activation-Associated Non-coding RNAs in Mammals. BMC Genomics 15 (1), 35. 10.1186/1471-2164-15-35 24438357 PMC3898825

[B107] Velichko A. K. Petrova N. V. Luzhin A. V. Strelkova O. S. Ovsyannikova N. Kireev I. I. (2019). Hypoosmotic Stress Induces R Loop Formation in Nucleoli and ATR/ATM-Dependent Silencing of Nucleolar Transcription. Nucleic Acids Res. 47 (13), 6811–6825. 10.1093/nar/gkz436 31114877 PMC6648358

[B108] Vera M. Biswas J. Senecal A. Singer R. H. Park H. Y. (2016). Single-Cell and Single-Molecule Analysis of Gene Expression Regulation. Annu. Rev. Genet. 50 (November), 267–291. 10.1146/annurev-genet-120215-034854 27893965 PMC5149423

[B109] Vieira-Vieira C. H. Selbach M. Selbach. Matthias. (2021). Opportunities and Challenges in Global Quantification of RNA-Protein Interaction via UV Cross-Linking. Front. Mol. Biosci. 8 (May). 10.3389/fmolb.2021.669939 PMC8155585 34055886

[B110] Vihervaara A. Duarte F. M. Lis J. T. (2018). Molecular Mechanisms Driving Transcriptional Stress Responses. Nat. Rev. Genet. 19 (6), 385–397. 10.1038/s41576-018-0001-6 29556092 PMC6036639

[B111] Vydzhak O. Luke B. Schindler N. (2020). Non-coding RNAs at the Eukaryotic rDNA Locus: RNA-DNA Hybrids and beyond. J. Mol. Biol. 432 (15), 4287–4304. 10.1016/j.jmb.2020.05.011 32446803

[B112] Wahba L. Costantino L. Tan F. J. Zimmer A. Koshland D. (2016). S1-DRIP-Seq Identifies High Expression and PolyA Tracts as Major Contributors to R-Loop Formation. Genes Dev. 30 (11), 1327–1338. 10.1101/gad.280834.116 27298336 PMC4911931

[B113] Wang A. H.-J. Fujii S. van Boom J. H. van der Marel G. A. van Boeckel S. A. A. Rich A. (1982). Molecular Structure of r(GCG)D(TATACGC): a DNA-RNA Hybrid helix Joined to Double Helical DNA. Nature 299 (5884), 601–604. 10.1038/299601a0 6181416

[B114] Wang I. X. Grunseich C. Fox J. Burdick J. Zhu Z. Ravazian N. (2018). Human Proteins that Interact with RNA/DNA Hybrids. Genome Res. 28 (9), 1405–1414. 10.1101/gr.237362.118 30108179 PMC6120628

[B115] Wang Z. Li K. Huang W. (2020). Long Non-coding RNA NEAT1-Centric Gene Regulation. Cell. Mol. Life Sci. 77 (19), 3769–3779. 10.1007/s00018-020-03503-0 32219465 PMC11104955

[B116] Wehner S. Dörrich A. K. Ciba P. Wilde A. Marz M. (2014). PRNA. RNA Biol. 11 (1), 3–9. 10.4161/rna.27448 24440945 PMC3929421

[B117] Weinreb J. T. Ghazale N. Pradhan K. Gupta V. Potts K. S. Tricomi B. (2021). Excessive R-Loops Trigger an Inflammatory Cascade Leading to Increased HSPC Production. Dev. Cel 56 (5), 627–640.e5. 10.1016/j.devcel.2021.02.006 PMC8258699 33651979

[B118] Wu T. Nance J. Chu F. Fazzio T. G. (2021). Characterization of R-Loop-Interacting Proteins in Embryonic Stem Cells Reveals Roles in rRNA Processing and Gene Expression. Mol. Cell Proteomics 20 (August), 100142. 10.1016/j.mcpro.2021.100142 34478875 PMC8461376

[B119] Wulfridge P. Sarma K. (2021). A Nuclease- and Bisulfite-Based Strategy Captures Strand-specific R-Loops Genome-wide. ELife 10 (February), e65146. 10.7554/eLife.65146 33620319 PMC7901872

[B120] Xiao S. Huang Q. Ren H. Yang M. (2021). The Mechanism and Function of Super Enhancer RNA. Genesis 59 (5–6), e23422. 10.1002/dvg.23422 34028961

[B121] Xu C. Wu Z. Duan H.-C. Fang X. Jia G. Dean C. (2021). R-loop Resolution Promotes Co-transcriptional Chromatin Silencing. Nat. Commun. 12 (1), 1790. 10.1038/s41467-021-22083-6 33741984 PMC7979926

[B122] Xu W. Li K. Li S. Hou Q. Zhang Y. Liu K. (2020). The R-Loop Atlas of Arabidopsis Development and Responses to Environmental Stimuli. Plant Cell 32 (4), 888–903. 10.1105/tpc.19.00802 32075864 PMC7145480

[B123] Xu W. Xu H. Li K. Fan Y. Liu Y. Yang X. (2017). The R-Loop Is a Common Chromatin Feature of the Arabidopsis Genome. Nat. Plants 3 (9), 704–714. 10.1038/s41477-017-0004-x 28848233

[B124] Yan Q. Sarma K. (2020). MapR: A Method for Identifying Native R‐Loops Genome Wide. Curr. Protoc. Mol. Biol. 130 (1), e113. 10.1002/cpmb.113 31943854 PMC6986773

[B125] Yang H. Berry S. Olsson T. S. G. Hartley M. Howard M. Dean C. (2017). Distinct Phases of Polycomb Silencing to Hold Epigenetic Memory of Cold in *Arabidopsis* . Science 357 (6356), 1142–1145. 10.1126/science.aan1121 28818969

[B139] Yaoyi L. Yawei S. Wei X. Qin L. Xinxiu W. Kuan L. (2020). R-loops coordinate with SOX2 in regulating reprogramming to pluripotency, Sci Adv. 2020 Jun 10; 6(24): eaba0777. 10.1126/sciadv.aba0777 32704541 PMC7360481

[B126] Yap K. Chung T. H. Makeyev E. V. (2022). Hybridization-Proximity Labeling Reveals Spatially Ordered Interactions of Nuclear RNA Compartments. Mol. Cel 82 (0), 463–478. 10.1016/j.molcel.2021.10.009 PMC8791277 34741808

[B127] Yi W. Li J. Zhu X. Wang X. Fan L. Sun W. (2020). CRISPR-assisted Detection of RNA-Protein Interactions in Living Cells. Nat. Methods 17 (7), 685–688. 10.1038/s41592-020-0866-0 32572232

[B138] Yu K. Chedin F. Hsieh C.-L. Wilson T. E.. Lieber M. R. (2003). R-Loops At Immunoglobulin Class Switch Regions In The Chromosomes Of Stimulated B Cells. Nat. Immunol. 4 (5), 442–451. 10.1038/ni919 12679812

[B128] Zeng C. Onoguchi M. Hamada M. (2021). Association Analysis of Repetitive Elements and R-Loop Formation across Species. Mobile DNA 12 (January), 3. 10.1186/s13100-021-00231-5 33472695 PMC7818932

[B129] Zhang T. Wallis M. Petrovic V. Challis J. Kalitsis P. Hudson D. F. (2019). Loss of TOP3B Leads to Increased R-Loop Formation and Genome Instability. Open Biol. 9 (12), 190222. 10.1098/rsob.190222 31795919 PMC6936252

[B130] Zhao J. Ohsumi T. K. Kung J. T. Ogawa Y. Grau D. J. Sarma K. (2010). Genome-Wide Identification of Polycomb-Associated RNAs by RIP-Seq. Mol. Cel 40 (6), 939–953. 10.1016/j.molcel.2010.12.011 PMC3021903 21172659

[B131] Zhao Z. Dammert M. A. Grummt I. Bierhoff H. (2016b). LncRNA-Induced Nucleosome Repositioning Reinforces Transcriptional Repression of RRNA Genes upon Hypotonic Stress. Cel Rep. 14 (8), 1876–1882. 10.1016/j.celrep.2016.01.073 26904956

[B132] Zhao Z. Dammert M. A. Hoppe S. Bierhoff H. Grummt I. (2016a). Heat Shock Represses RRNA Synthesis by Inactivation of TIF-IA and LncRNA-dependent Changes in Nucleosome Positioning. Nucleic Acids Res. 44 (17), 8144–8152. 10.1093/nar/gkw496 27257073 PMC5041454

[B133] Zhao Z. Sentürk N. Song C. Grummt I. (2018). Lncrna Papas Tethered to the Rdna Enhancer Recruits Hypophosphorylated Chd4/Nurd to Repress Rrna Synthesis at Elevated Temperatures. Genes Dev. 32 (11–12), 836–848. 10.1101/gad.311688.118 29907651 PMC6049515

